# Uterine cyclin A2–deficient mice as a model of female early pregnancy loss

**DOI:** 10.1172/JCI163796

**Published:** 2024-09-12

**Authors:** Fatimah Aljubran, Katelyn Schumacher, Amanda Graham, Sumedha Gunewardena, Courtney Marsh, Michael Lydic, Kristin Holoch, Warren B. Nothnick

**Affiliations:** 1 Department of Cell Biology and Physiology,; 2 Department of Obstetrics and Gynecology,; 3 Center for Reproductive Sciences,; 4 Department of Cancer Biology,; 5 Institute for Reproductive and Developmental Sciences, University of Kansas Medical Center, Kansas City, Kansas, USA.

**Keywords:** Reproductive biology, Fertility, Obstetrics/gynecology, Sex hormones

## Abstract

Proper action of the female sex steroids 17β-estradiol (E2) and progesterone (P4) on the endometrium is essential for fertility. Beyond its role in regulating the cell cycle, cyclin A2 (CCNA2) also mediates E2 and P4 signaling in vitro, but a potential role in modulating steroid action for proper endometrial tissue development and function is unknown. To fill this gap in our knowledge, we examined human endometrial tissue from fertile and infertile cisgender women for CCNA2 expression and correlated this with pregnancy outcome. Functional assessment of CCNA2 was validated in vivo using a conditional Ccna2 uterine-deficient mouse model, while in vitro function was assessed using human cell culture models. We found that CCNA2 expression was significantly reduced in endometrial tissue, specifically the stromal cells, from women undergoing in vitro fertilization who failed to achieve pregnancy. Conditional deletion of Ccna2 from mouse uterine tissue resulted in an inability to achieve pregnancy, which appeared to be due to alterations in the process of decidualization, which was confirmed using in vitro models. From these studies, we conclude that CCNA2 expression during the proliferative/regenerative stage of the menstrual cycle allows for proper steroid responsiveness, decidualization, and pregnancy. When CCNA2 expression levels are insufficient, there is impaired endometrial responsiveness, aberrant decidualization, and loss of pregnancy.

## Abstract 2

Cyclin A2 expression during the proliferative/regenerative stage of the menstrual cycle is essential for proper steroid responsiveness, decidualization and pregnancy.

## Graphical abstract

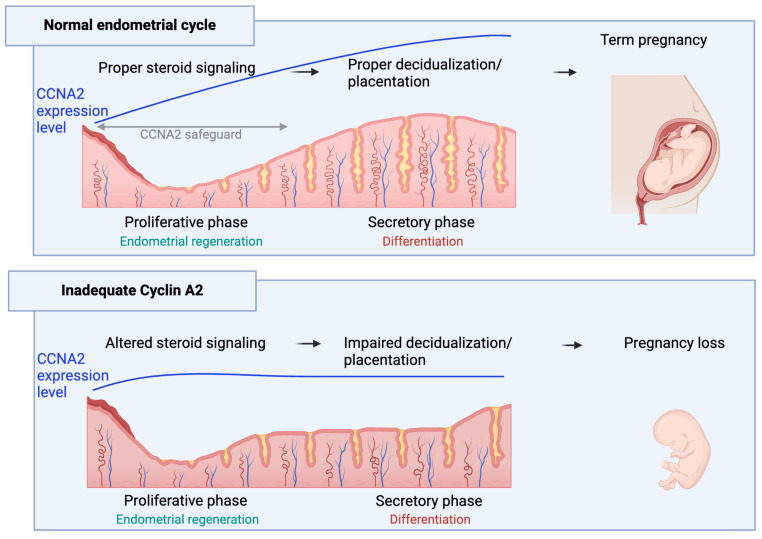

## Introduction

Infertility is a major public health problem that affects 19% of reproductive-age women in the US (1). Common categories of female infertility include ovulatory disorders, tubal occlusion, and uterine disorders (2). However, the spectrum of female infertility extends to include pregnancy disorders that lead to the loss of the embryo, such as implantation failure, miscarriages, or pregnancy loss. Implantation failure or the absence of clinical pregnancy accounts for 50% of infertility cases (3). On the other hand, pregnancy loss is defined as the loss of the fetus before 20 weeks of gestation, which affects 10%–15% of pregnant women (4). Growing evidence suggests that abnormal uterine function plays a major role in implantation failure and pregnancy loss (3, 5). For instance, defective decidualization has been implicated in poor pregnancy outcomes, such as recurrent pregnancy loss (RPL) and preeclampsia (6–8).

The uterus provides a niche for the embryo to implant and grow through an intricate system that connects the uterine endometrium with the embryonic tissue known as the placenta. This process requires dynamic remodeling of the uterine lining to accommodate the implanting embryo. Moreover, cyclical remodeling of the endometrium is greatly dependent upon steroid hormone action mediated by steroid receptors.

Decidualization is the transformation of endometrial stromal fibroblasts into specialized secretory, decidual cells and is essential for embryo implantation, placentation, and successful pregnancy. Not surprisingly, defective stromal cell decidualization has been linked to pregnancy complications in women, including pregnancy loss and miscarriage (7). Unfortunately, our understanding of how defective stromal cell decidualization leads to pregnancy loss is poor. Studies that have examined decidualization and pregnancy establishment have primarily utilized samples from the secretory stage of the menstrual cycle and/or the “window of implantation.” While this approach is logical and outcomes have undoubtedly provided important information on mediators and mechanisms required for proper decidualization and pregnancy, assessment of only the latter half of a menstrual cycle may limit a full understanding of the events that are necessary for proper decidualization. The necessity for proper endometrial preconditioning, tissue regeneration, and proliferation that occurs in the first half of a menstrual cycle for successful pregnancy has been proposed (9, 10), but few, if any, studies have mechanistically explored this postulate.

Cyclins are regulatory proteins that initiate the entry and progression of the cell cycle through the activation of cyclin-dependent kinases (CDKs). Growing evidence suggests that cyclins and CDKs elicit noncanonical functions in diverse cellular models (11). Cyclins have been shown to regulate steroid receptor action in CDK-dependent and independent manners (12–16). Cyclin A2 (CCNA2) is predominantly expressed during the S-phase of the cell cycle and interacts with CDK1 and CDK2 to activate downstream targets and induce cell-cycle progression (11). CDKs are proline-directed kinases that phosphorylate proteins containing Ser/Thr-Pro motifs such as the steroid receptors and their coactivators (12, 13). The CCNA2/CDK2 complex has been shown to enhance estrogen receptor α (ERα) transcriptional activity in vitro through phosphorylation, specifically at serine residues 104 and 106 (13). Likewise, CCNA2/CDK2 phosphorylates progesterone (P4) receptor (PGR) and steroid receptor coactivator 1 (SRC1), leading to the potentiation of PGR transcriptional activity in vitro (12, 14–16).

Although the endometrium is a steroid-responsive tissue, the potential role of CCNA2 in endometrial function has not been explored. Given its ability to modulate steroid responsiveness in other cell types, we hypothesized that reduced expression of uterine CCNA2 may be associated with aberrant endometrial steroid signaling, leading to endometrial dysfunction and infertility. To better understand the potential role of CCNA2 in endometrial signaling, we created a conditional knockout mouse model using P4 receptor cre (*Pgr^cre^*) recombinase to mediate CCNA2 (*Ccna2*) deletion in the female reproductive tract. The present study reveals that CCNA2 deficiency in vivo and in vitro is associated with impaired endometrial responsiveness to steroid signaling, leading to pregnancy loss in utero that may be due to defective decidualization and/or placentation.

## Results

### Endometrial CCNA2 expression is reduced in women who fail to achieve IVF-assisted pregnancy.

Endometrial biopsies were obtained from subjects during the early proliferative (EP) or late proliferative (LP) stages of an ovarian stimulation protocol as defined in Supplemental Methods. Subjects undergoing in vitro fertilization (IVF) for advanced maternal age/diminished ovarian reserve, endometriosis, tubal factor infertility, RPL, unexplained infertility, or male factor infertility (Table 1) provided biopsies. Pregnancy outcomes were assessed 12 to 14 days after a future frozen embryo transfer by measuring serum β-human chorionic gonadotropin (β-hCG). A positive pregnancy was defined as a β-hCG value of greater than 5 IU/L, while less than 5 IU/L was considered a negative pregnancy outcome (Table 1).

**Figure 1:**
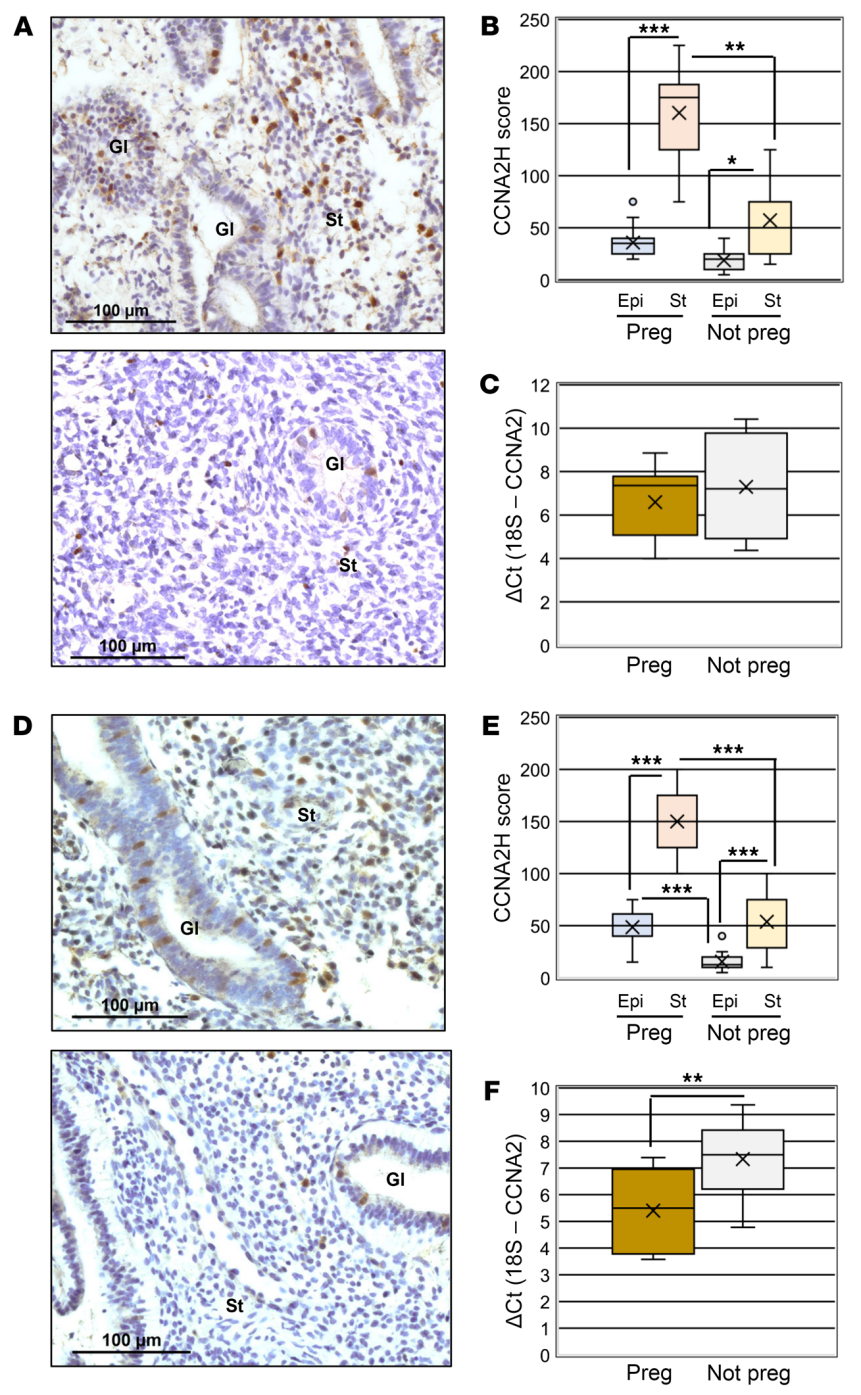
CCNA2 localization in early and LP endometrial biopsies from cisgender women who achieved or failed to achieve ART pregnancy. Endometrial biopsies were obtained from cisgender women who achieved pregnancy (Preg) and those that did not (Not Preg) during the (**A** and **B**) early or late (**D** and **E**) proliferative stage of a stimulation cycle. Scale bars: 100 μM. Gl, endometrial gland; St, stroma. H-scores were calculated for CCNA2 expression in early (**B**) and late (**D**) proliferative biopsies. qRT-PCR was performed for *CCNA2* on sections from the same blocks for which IHC was performed and expressed as ΔCt values and FC in *CCNA2* expression for early (**C**, *n* = 7, Preg; *n* = 4, Not Preg) and late proliferate stage samples (**F**, *n* = 14, Preg; *n* = 10, Not Preg). H-Score data were analyzed by 1-way ANOVA and post-hoc analysis within cycle stage among the 4 groups with *P* values indicated. FC in *CCNA2* mRNA (**C** and **F**) was analyzed by 2-tailed unpaired *t* test. *n* = 17, EP Preg; *n* = 13, EP Not Preg; *n* = 14, LP Preg; *n* = 12, LP Not Preg. Within each graph of the box and whisker plot, X indicates the mean values while the whisker end points indicate the highest and lowest values; circles represent outliers which are defined as more than 1.5 times the interquartile range away from the box. *P < 0.05; **P < 0.01; ***P < 0.001.

In EP specimens from subjects who achieved a positive pregnancy, CCNA2 expression was predominantly within the stroma, showing strong nuclear staining compared with specimens from subjects who had a negative pregnancy test (Figure 1A). Assessment of H-scores revealed that stromal cell expression of CCNA2 was higher in endometrial biopsies from cisgender women who achieved pregnancy compared with those that did not (Figure 1B). Further, in EP specimens, stromal cell expression was greater compared with epithelium in subjects who achieved pregnancy as well as those that did not (Figure 1B). There was no difference in epithelial cell expression of CCNA2 between study groups (Figure 1B). Assessment of *CCNA2* transcript in serial sections from the same biopsies used for IHC localization revealed no difference in the level of mRNA expression between study groups (Figure 1C). A similar pattern of localization and expression was observed in endometrial biopsies collected during the LP time point (Figure 1, D and E), with CCNA2 expression being predominantly within the nucleus of stromal cells. However, unlike EP specimens, LP specimens expressed not only greater CCNA2 levels in the stromal cells of subjects who achieved pregnancy compared with those who did not (Figure 1E), but epithelial cell expression was also higher in these biopsies (Figure 1E). Also, in contrast to EP specimens, *CCNA2* transcript expression was significantly greater in LP biopsies from subjects who achieved pregnancy (Figure 1F). To verify that these elevated levels of transcript expression were not due to an increase in epithelial cell content in these biopsies, we assessed cytokeratin 18 (*KRT18*) in these same RNA samples as previously described (17) and found no difference in Ct values (± SEM) between groups (LP pregnant = 6.79 ± 0.42 compared with 6.93 ± 0.43 for LP not pregnant; *P* = 0.824; *P* > 0.05).

Because CCNA2 plays a critical role in cellular growth and proliferation, we assessed endometrial cell proliferation in EP and LP endometrial biopsies among study groups. Immunostaining of Ki67 did not differ between subjects that achieved pregnancy and those that failed to achieve pregnancy in the EP group (Supplemental Figure 1A; supplemental material available online with this article; https://doi.org/10.1172/JCI163796DS1). However, during the LP stage, proliferation index increased in endometrial stroma compared with the epithelium in both groups, but only reaching statistical significance in cisgender women that achieved pregnancy (Supplemental Figure 1B). Because ERα and PGR mediate steroid hormone action in endometrial tissue and due to the potential role of CCNA2 in mediating these effects (12–16), we assessed the expression levels of ERα and PGR among subjects that achieved pregnancy and those that did not. In EP specimens, ERα expression was greater in stromal cells from subjects who achieved pregnancy compared with those who did not (Supplemental Figure 2A). Similarly, in LP specimens, stromal cell ERα expression was higher in the group that achieved pregnancy compared with those who did not (Supplemental Figure 2B). However, ERα expression was only greater in epithelial cells compared with stromal cell expression in the group that did not achieve pregnancy (Supplemental Figure 2B). PGR expression was not different in EP specimens for either epithelial or stromal cells between groups (Supplemental Figure 3A). In contrast, PGR expression was lower in stromal cells compared with epithelial cells from nonpregnant subjects and was also significantly lower in stromal cells compared with stromal cell expression in those subjects who achieved pregnancy (Supplemental Figure 3B). Collectively, these data suggest that reduced levels of CCNA2 expression in endometrial tissue from women who failed to achieve pregnancy was associated with reduced stromal cell proliferation (Ki67) as well as expression of ERα and PGR compared with those subjects who achieved pregnancy.

### Infertility is recapitulated in a conditional uterine knockout mouse model of Ccna2.

**Figure 2:**
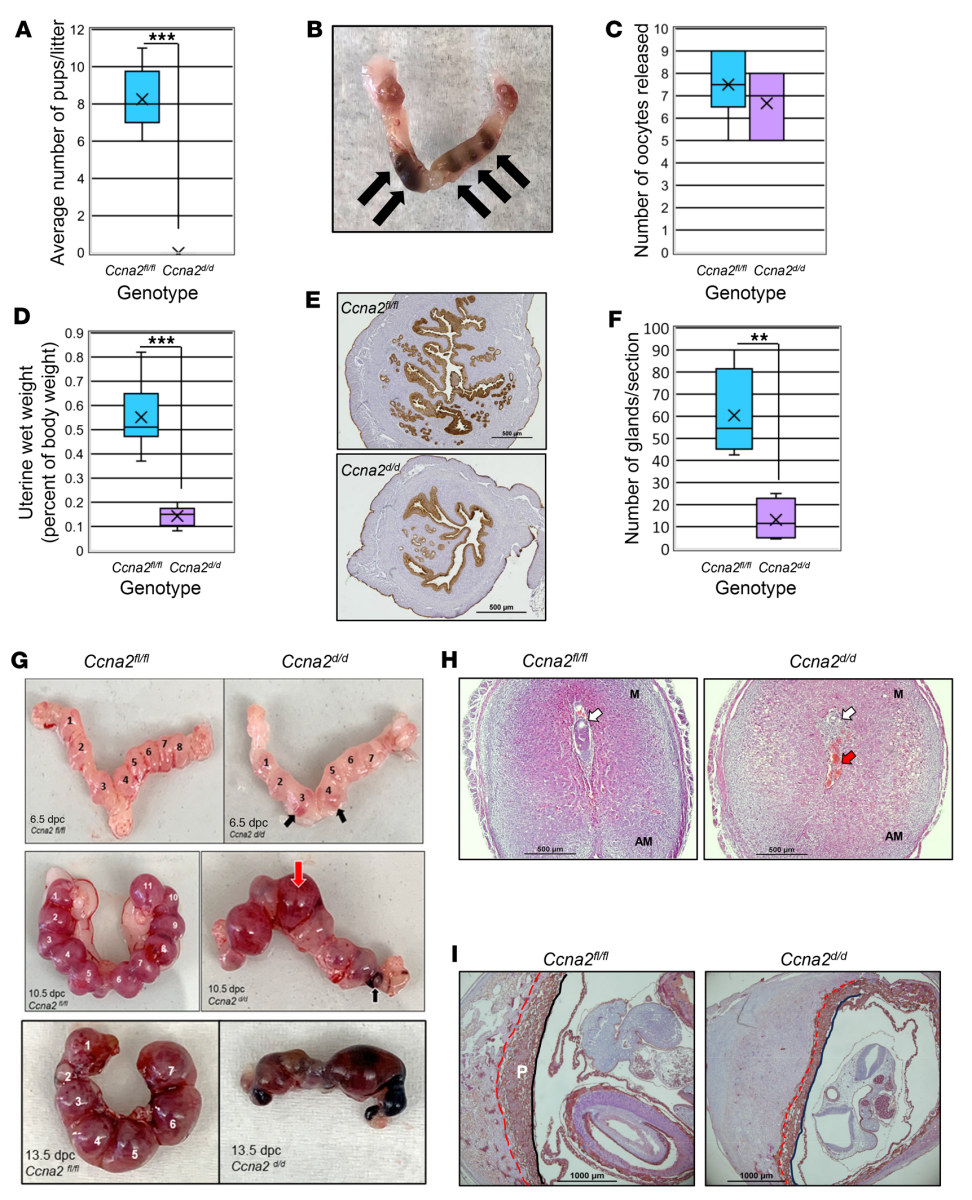
Deletion of uterine Ccna2 leads to pregnancy loss and uterine alterations. (**A**) Female mice in which Ccna2 is deleted from the uterus fail to give birth to offspring. At term (dpc 19.5), Ccna2-deficient mice (*Ccna2^d/d^*) exhibit fetal resorption (**B**, indicated by black arrows). *Ccna2^d/d^* released a similar number of oocytes at dpc 0.5, (**C**) but had significantly smaller uteri (**D**), which were assessed by Krt19 immunohistochemistry (**E**) and counting Krt19-positive glands/section (**F**). Assessment of implantation sites at dpc 6.5, 10.5, and 13.5 (**G**) revealed signs of hemorrhagic implantation sites and resorption of implantation sites between dpc 6.5 (black arrows; top panel) and dpc 10.5 (middle panel; red arrows = inflammation, black arrow = resorption) in Ccna2-knockout (*Ccna2^d/d^*) mice. (**H**) Histological assessment of dpc 6.5 implantation sites shows signs of decidualization, but loss of a viable embryo (white arrow) and accumulation of red blood cells (red arrow) in *Ccna2^d/d^* mice. M, mesometrial side; AM, antimesometrial side. (**I**) Immunohistochemical localization of cytokeratin 8 highlights abnormal placenta (P) in *Ccna2^d/d^* mice (outlined by red and black lines). *n* = 4–6 mice/genotype per observation for all data (panels **A**–**I**). Data in panels **A**, **C**, **D** and **F** were analyzed by 2-tailed unpaired *t* tests. **P < 0.01, ***P < 0.001. Within each graph of the box and whisker plot, X indicates the mean values while the whisker end points indicate the highest and lowest values. Scale bars: 500 μm (**E** and **H**); 1,000 μm (**I**).

Data from our human study suggested a direct relationship between CCNA2 expression and pregnancy outcome. To determine whether reduction/loss of CCNA2 expression functionally contributes to the ability to establish and maintain pregnancy, we developed a mouse model in which *Ccna2* was conditionally deleted from the female reproductive tract (*Ccna2^d/d^*) using Pgr^Cre^ mice to delete *Ccna2* expression within the uterus. Female mice that expressed *Ccna2* (*Ccna2^fl/fl^*) or had the gene deleted (*Ccna2^d/d^*) were mated with WT (C57BL/6) males of proven fertility. Females of both genotypes mated (as evidenced by a copulatory plug which is considered day post coitum [dpc] 0.5), but only *Ccna2^fl/fl^* gave birth to pups (Figure 2A). Necropsy of *Ccna2^d/d^* females at dpc 19.5 revealed the presence of resorbed fetuses (Figure 2B) in all *Ccna2^d/d^* females.

To begin to evaluate where in the reproductive process fetal loss may occur, we mated a separate cohort of *Ccna2^fl/fl^* and *Ccna2^d/d^* females with WT males of proven fertility and sacrificed females at dpc 0.5 (peri-ovulatory period), 6.5 (post-embryo implantation, decidualization), 10.5 (placentation), and 13.5 (post-placentation). In the dpc 0.5 groups, we first assessed ovulation rate to verify that a similar number of oocytes were released. As depicted in Figure 2C, mice of both genotypes released an average of approximately 7 oocytes. Upon harvesting the female reproductive tract to assess ovulated oocytes, it became evident that uteri from the *Ccna2^d/d^* females were considerably smaller and lacked vascularity compared with *Ccna2^fl/fl^* uteri. Assessment of uterine wet weight revealed that *Ccna2^d/d^* uteri weighed approximately one-fifth of that of *Ccna2^fl/fl^* females (Figure 2D) and were characterized by fewer endometrial glands (assessed by Krt19 staining; Figure 2E) compared with *Ccna2^fl/fl^* uteri (Figure 2F). Mice of both genotypes exhibited a similar number of embryo implantation sites (numbered in Figure 2G), but those implantation sites in *Ccna2^d/d^* females began to exhibit signs of hemorrhage (Figure 2G), which became more apparent at midterm pregnancy (dpc 10.5; Figure 2G) with fetal loss apparent at dpc 13.5 (Figure 2G). Histological assessment of implantation sites at dpc 6.5 indicated that embryo loss was beginning to occur in the *Ccna2^d/d^* mice (Figure 2H) and that at dpc 10.5 (Figure 2I), placenta development was compromised. These alterations in the uterus and pregnancy maintenance could not be attributed to insufficient systemic levels of 17β-estradiol (E2) (Supplemental Figure 4A) or altered patterns of localization and levels of Erα expression at dpc 0.5 (Supplemental Figure 4B). Further, serum P4 concentrations at dpc 6.5 (Supplemental Figure 4C) or dpc 10.5 (Supplemental Figure 4D) as well as prolactin levels (Supplemental Figure 4E) at dpc 10.5 did not differ between mice of either genotype. In fact, at dpc 10.5, serum P4 and prolactin levels were actually higher in the Ccna2-deficient mice compared with *Ccna2^fl/fl^* counterparts (Supplemental Figure 4, D and E). It is possible that the elevated P4 levels in the *Ccna2^d/d^* mice could contribute to impaired pregnancy, as Liang and colleagues (18) reported that mice treated with increasing P4 doses experienced impaired decidualization in vivo.

### Loss of uterine Ccna2 is associated with an altered matrisome.

**Figure 3:**
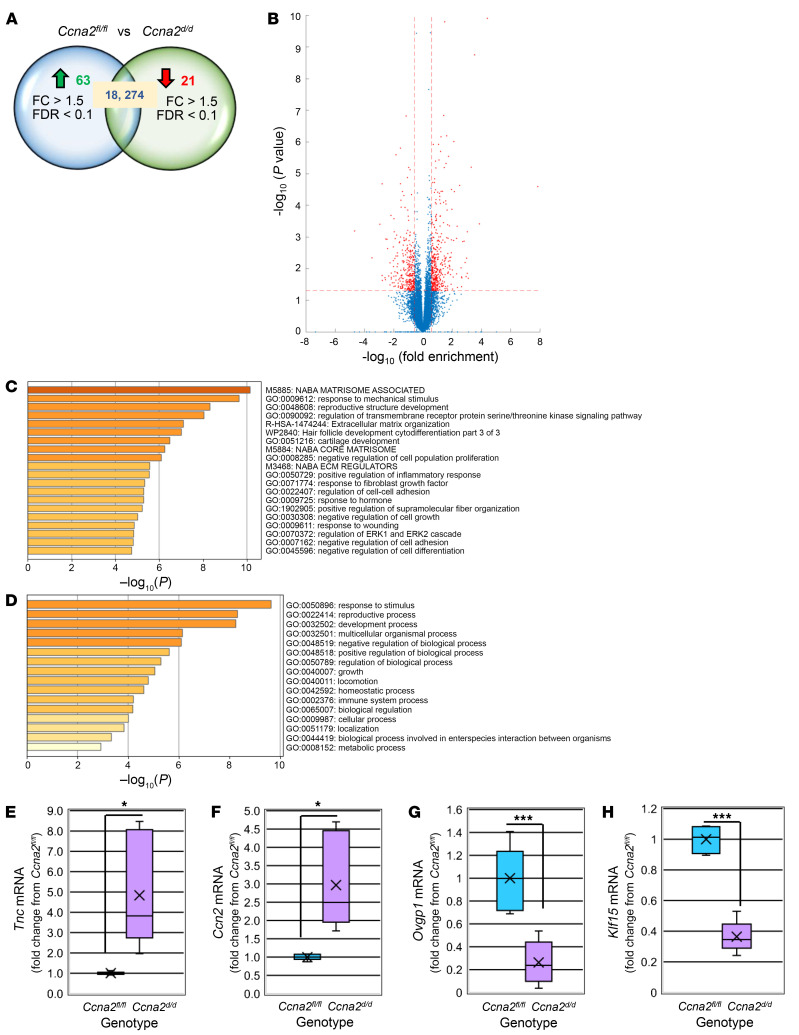
Bulk RNA-Seq analysis in dpc 0.5 uteri. (**A**) Venn diagram and (**B**) volcano plot shows the number and distribution of DEGs in dpc 0.5 uteri of *Ccna2^d/d^* mice (*P* < 0.05). (**C**) Pathway and (**D**) process analysis for the DEGs (>1.5 FC, *P* < 0.05, *q* < 0.1) in dpc 0.5 uterine tissue between genotypes. (**E**–**H**) FC in 2 of the top upregulated and downregulated genes by RNA-Seq analysis and validation by qRT-PCR for (**E**) *Tnc*, (**F**) *Ccn2*, (**G**) *Ovgp1*, and (**H**) *Klf15*. RNA was isolated from *n* = 4 mice/genotype used to generate the volcano plot and from *n* = 6 mice/genotype for qRT-PCR validation studies. Data were analyzed by 2-tailed unpaired *t* tests for qRT-PCR data between genotypes (panels **E**–**H**). *P < 0.05; *** *P* < 0.001. Within each graph of the box and whisker plot, X indicates the mean values while the whisker end points indicate the highest and lowest values.

To begin to assess the mechanisms by which *Ccna2* deficiency prior to embryo implantation may contribute to pregnancy loss, we performed bulk RNA-Seq on dpc 0.5 uteri from mice of both genotypes to characterize the uterine transcriptome prior to the embryonic decidual signal. A total of 18,270 genes were identified in uterine tissue at dpc 0.5 (Figure 3, A and B). Using a fold change (FC) of ≥1.5, a *P* < 0.05, and a false discovery rate (FDR) of < 0.1, 63 genes were upregulated (Supplemental Table 1) and 21 were downregulated (Supplemental Table 2) in dpc 0.5 uteri from *Ccna2^d/d^* mice. Pathway (Figure 3C) and process (Figure 3D) enrichment analysis revealed the top-level Gene Ontology biological processes and top 20 clusters with their representative enriched terms. Interestingly, *Ccna2^d/d^* uterine tissue exhibited altered expression of key components of the matrisome, which is an ensemble of extracellular matrix (ECM) and ECM-associated proteins that includes ECM proteins, proteases, and cytokines, capable of regulating multiple cellular processes (19, 20). Focusing on the top differentially expressed genes (DEGs) (Supplemental Tables 1 and 2) using cutoff values of greater than 2 FC between genotypes, *P* < 0.05 and FDR < 0.1, we validated 2 of the most substantially upregulated and 2 of the most considerably downregulated transcripts associated with the ECM/matrisome. These upregulated transcripts were identified as tenascin (*Tnc*) (4.3-fold; Figure 3E) and cellular communication network factor 2 (*Ccn2*) (2.63-fold; Figure 3F), which is also known as connective tissue growth factor (*Ctgf*), while the downregulated transcripts were identified as oviductal glycoprotein 1 (*Ovgp1*) (4.56-fold; Figure 3G) and Kruppel like factor 15 (*Klf15*) (2.9-fold; Figure 3H). Interestingly, all 4 of these transcripts have been reported to be associated with embryo implantation and decidualization (21–34). In addition, the majority of the downregulated genes (Supplemental Table 2) were determined to be epithelial cell enriched, which may reaffirm the necessity of glandular epithelial-derived factors for proper endometrial decidualization in both mouse (35–37) and humans (38).

### Loss of uterine Ccna2 is associated with impaired E2 action and steroid signaling.

**Figure 4:**
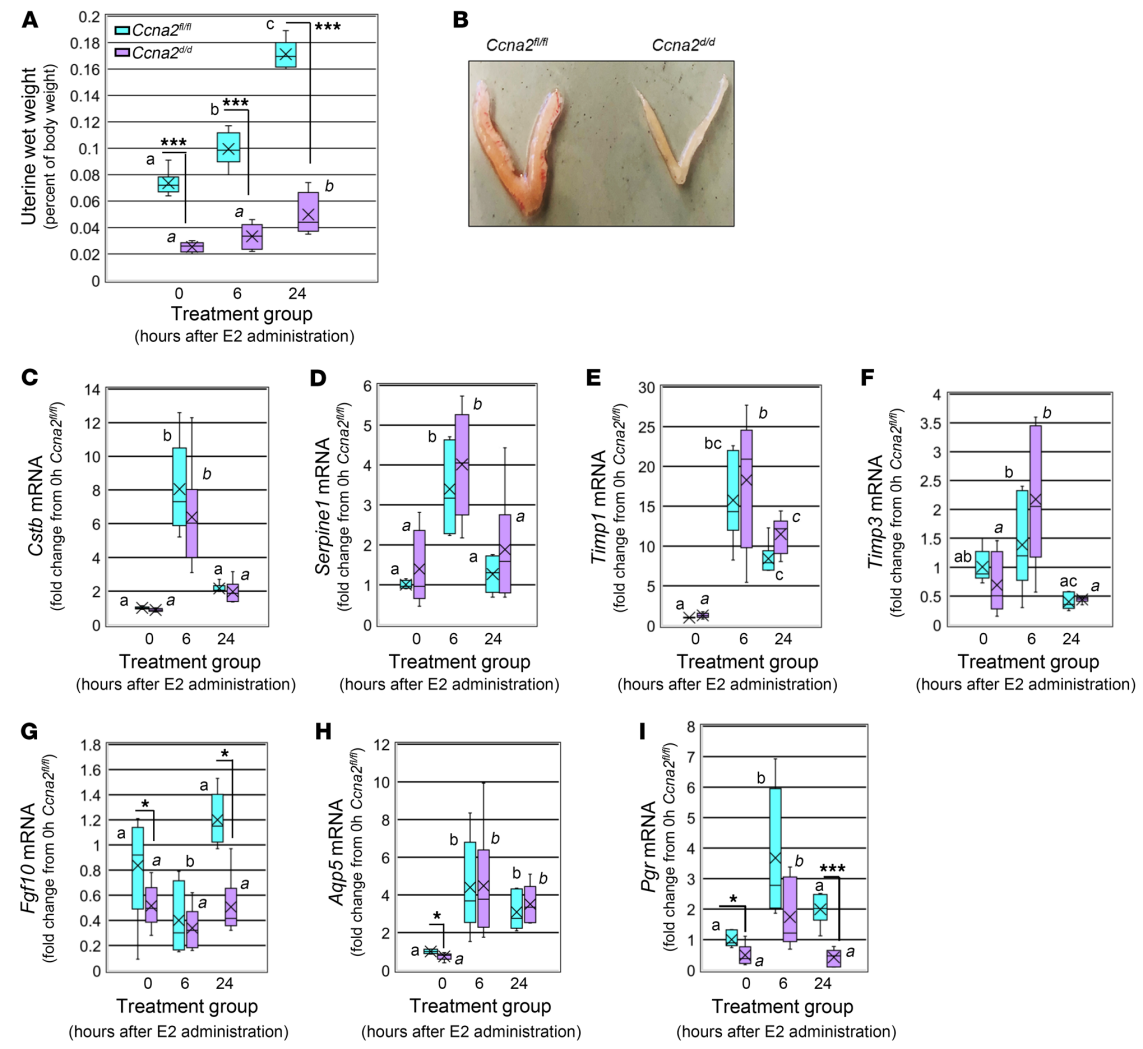
Select impaired estrogen action in Ccna2^d/d^ uteri. (**A**) Uterine wet weight at 0 hours (prior to treatment), 6, and 24 hours after E2 treatment. Different letters indicate statistical significance among the means for the treatment groups within treatment groups by 1-way ANOVA (block letters compare *Ccna2^fl/fl^* and italicized letters compare *Ccna2^d/d^*). ***P < 0.001 between genotypes within each treatment group (*n* = 6 mice/group). In **A**, a versus b = *P* < 0.05, a versus c = *P* < 0.01, b versus c = *P* < 0.05; *a* versus *b* = *P* < 0.05. (**B**) Gross uterine morphology and histology in *Ccna2^fl/fl^* and *Ccna2^d/d^* mice at 24 hours after E2 treatment. (**C**–**H**) E2 regulation of (**C**) *Cstb*, (**D**) *Serpine1*, (**E**) *Timp1*, (**F**) *Timp3*, (**G**) *Fgf10*, (**H**) *Aqp5*, and (**I**) *Pgr* transcripts between genotypes. Different block letters indicate statistical significance among the means for the *Ccna2^fl/fl^* treatment groups, while different letters in italics indicate statistically significant differences among the means in *Ccna2^d/d^* mice by 1-way ANOVA. In **C** and **D**, a versus b = *P* < 0.001, *a* versus *b* = *P* < 0.001; in **E**, a versus bc = *P* < 0.001, *a* versus *b* = *P* < 0.001; *a* versus *c* = *P* < 0.05, *b* versus *c* = *P* < 0.05; in **F**, *a* versus *b* = *P* < 0.05; in **G**, a versus b = *P* < 0.05; in **H**, a versus b = *P* < 0.05, *a* versus *b* = *P* < 0.05; in **I,** a versus b = *P* < 0.05, *a* versus *b* = *P* < 0.05. *P < 0.05; ***P < 0.001 between genotypes within each treatment group (*n* = 6 mice/group) by 2-tailed unpaired *t* test between genotypes. Within each graph of the box and whisker plot, X indicates the mean values while the whisker end points indicate the highest and lowest values.

To determine whether the lower uterine wet weight characteristic of *Ccna2^d/d^* mice (Figure 2D) could be attributed to altered steroid action, female mice of both genotypes were ovariectomized and treated with E2. Uterine wet weight of *Ccna2^d/d^* mice was lower compared with *Ccna2^fl/fl^* in all treatment groups (Figure 4A), being about one-third the uterine wet weight of control counterparts at all 3 time points. Compared with the control, untreated (0 hours) group, *Ccna2^fl/fl^* mice treated with E2 gained uterine wet weight at 6 hours (1.36-fold increase over 0 hours) and 24 hours (2.31-fold increase over 0 hours), which was statistically significant at 24 hours within genotype (Figure 4A). Although E2-treated *Ccna2^d/d^* mice did exhibit a marked increase in uterine wet weight at 24 hours compared with 0 hours, this increase was substantially lower compared with that of the *Ccna2^fl/fl^* group at 24 hours (Figure 4A). It should be noted, however, that within genotype, the fold increases over the 0 hours group in the *Ccna2^d/d^* mice were 1.31- and 1.96-fold at 6 and 24 hours, respectfully, which was very similar to increases observed in the *Ccna2^fl/fl^* mice. In addition to the lower uterine wet weights, uteri of *Ccna2^d/d^* mice appeared less vascular and smaller from both gross and histological assessment (Figure 4B). To further evaluate hormonal responsiveness of uteri from *Ccna2^d/d^*, we assessed the expression of known E2-regulated genes from mice of both genotypes.

While many of these genes increased in response to E2 treatment, there were no differences in their levels of expression between genotypes. More specifically, transcript expression of cystatin B (*Cstb*) (Figure 4C) and serpine1 (*Serpine1*) (Figure 4D), which are, respectively, cysteine and serine protease inhibitors, was increased by E2 at 6 hours after administration, returning to control/baseline levels at 24 hours. There was no difference in the pattern or extent of induction of these protease inhibitors between genotypes at any of the time points, including 0 hours. Tissue inhibitors of metalloproteinases, *Timp1* (Figure 4E) and *Timp3* (Figure 4F), also exhibited a marked increase in expression at 6 hours after E2 administration. E2 increased *Timp1* expression in mice of both genotypes at 6 and 24 hours. In contrast, *Timp3* levels only showed different levels of expression in the *Ccna2^fl/fl^* uteri at 6 hours compared with 24 hours. On the contrary, E2 induced an increase in *Timp3* expression at 6 hours in *Ccna2^d/d^* uteri, which returned to baseline levels at 24 hours. Like *Ctsb* and *SerpinE1*, there were no differences in the extent of induction of either *Timp1* or *Timp3* by E2 between genotypes.

Assessment of fibroblast growth factor-10 (*Fgf10*) (Figure 4G) and aquaporin-5 (*Aqp5*) (Figure 4H), which may contribute to endometrial stromal cell proliferation and uterine water inhibition, respectively, revealed slightly different patterns of expression. Compared with 0 hours, *Fgf10* transcript expression in *Ccna2^fl/fl^* was substantially reduced at 6 hours after E2 administration and returned to baseline/control levels at 24 hours (Figure 4G). In contrast, *Fgf10* expression in *Ccna2^d/d^* uterine tissue did not respond to E2 treatment, as expression was lower compared with *Ccna2^fl/fl^* tissue levels at 0 hours and 24 hours. E2 increased *Aqp5* at both 6 and 24 hours after administration and the levels of expression did not differ between genotypes at these time points (Figure 4G). At 0 hours, *Aqp5* expression was lower in 0 hours uterine tissue in *Ccna2^d/d^* mice compared with controls (Figure 4H). Of interest was the observation that both *Fgf10* and *Aqp5* transcript expression was lower in dpc 0.5 uteri from *Ccna2^d/d^* mice compared with *Ccna2^fl/fl^* mice (Figure 4, G and H), which may suggest that these genes may be disrupted early during uterine development and may contribute to the smaller uteri detected in the *Ccna2^d/d^* mice. Given that *PGR* is an E2-responsive gene and PGR expression was reduced in endometrium of subjects who failed to achieve pregnancy (Supplemental Figure 3B), we also assessed *Pgr* expression in response to E2 treatment. In the 0-hours groups, *Pgr* expression was higher in *Ccna2^fl/fl^* mice compared with *Ccna2^d/d^* mice (Figure 4I). E2 increased *Pgr* expression in *Ccna2^fl/fl^* mice (*P* < 0.05) ranging from approximately 2- to 6-fold above 0 hour levels, while the increase in *Ccna2^d/d^* ranged from approximately 1.2- to 3-fold above 0 hour controls (Figure 4I). At 24 hours after E2 treatment, *Ccna2^d/d^* mice exhibited significantly lower levels of *Pgr* transcript expression compared with controls (Figure 4I).

### Impaired estrogen response at dpc 0.5 in Ccna2-deficient mice is associated with reduced expression of phospho-Ser104-estrogen receptor.

**Figure 5:**
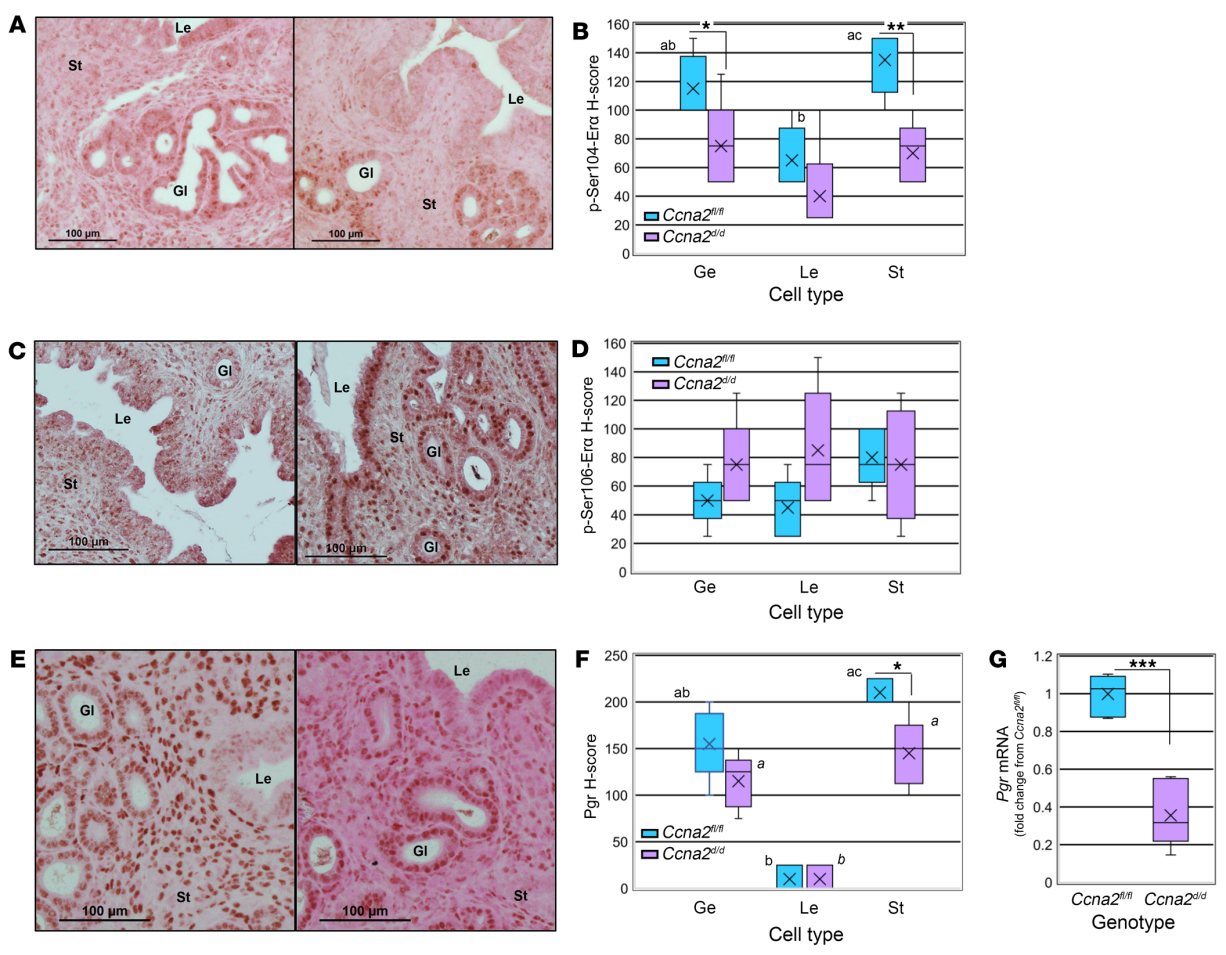
Loss of uterine Ccna2 alters phosphorylation of estrogen receptor. (**A**) Immunohistochemical localization and (**B**) semiquantitation of phospho-ser104-Erα expression in uterine tissue of *Ccna2^fl/fl^* and *Ccna2^d/d^* mice at dpc 0.5. (**C**) Immunohistochemical localization and (**D**) semiquantitation of phospho-ser106-Erα expression in uterine tissue of *Ccna2^fl/fl^* and *Ccna2^d/d^* mice at dpc 0.5. (**E**) Immunohistochemical localization, (**F**) semiquantitation of Pgr expression, and (**G**) assessment of *Pgr* transcript in uterine tissue of *Ccna2^fl/fl^* and *Ccna2^d/d^* mice at dpc 0.5. Within each graph of the box and whisker plot, X indicates the mean values while the whisker end points indicate the highest and lowest values. H-score data were analyzed by 1-way ANOVA among cell types followed by Bonferroni’s multiple planned comparisons within genotype among cell types. Different letters indicate statistical significance among the means within genotype for *Ccna2^fl/fl^* (block letters) and *Ccna2^d/d^* (italicized letters). In **B**, b versus ac = *P* < 0.05; in **F**, b versus ac = *P* < 0.01, *a* versus *b* and *b* versus *a* = *P* < 0.05. *P < 0.05; **P < 0.01; ***P < 0.001. Le, lumen. Data are representative of *n* = 5 mice/genotype for IHC data and *n* = 6 mice/genotype for *Pgr* data.

As CCNA2-CDK2 phosphorylates Erα at serine (ser) residues 104 and 106 (13), we wished to examine their localization in dpc 0.5 uteri from Ccna2-deficient mice. Compared with *Ccna2^fl/fl^* controls, *Ccna2^d/d^* expression of phospho-ser104-Erα was lower in both glandular epithelium and stromal cells, while luminal epithelial cell expression was similarly low in uteri from mice of both genotypes (Figure 5, A and B). Expression of phospho-ser106-Erα was more variable than that of phospho-ser104-Erα and showed similar levels of expression between genotypes (Figure 5, C and D). As it is well established that E2 induces expression of Pgr, we evaluated its localization in dpc 0.5 uterine sections. Compared with controls, *Ccna2^d/d^* mice exhibited lower levels of Pgr expression in stromal cells while expression in epithelial cells was similar between genotypes (Figure 5, E and F) and this lower expression was also confirmed on whole uterine tissue at dpc 0.5 by quantitative reverse-transcription PCR (qRT-PCR) of *Pgr* transcript (Figure 5G).

### CCNA2 deficiency impairs E2 induction of stromal cell P4 receptor expression in vitro.

To further examine the reduced expression of stromal cell *PGR* expression, we knocked down CCNA2 expression in human stromal cells (t-HESC; ref. 39) and subsequently treated them with E2 for 6 hours, then assessed *PGR* expression by qRT-PCR. Knockdown of CCNA2 was associated with a marked reduction of PGR expression compared with cells transfected with a nontargeting (NT) siRNA (Supplemental Figure 5A). Knockdown of *CCNA2* by CCNA2 siRNA was confirmed by qRT-PCR in these same samples (Supplemental Figure 5B). Thus, deletion/reduction of CCNA2 in both human and mouse models resulted in reduced E2 induction of PGR expression.

### Loss of uterine Ccna2 is associated with altered decidualization and placentation in vivo.

**Figure 6:**
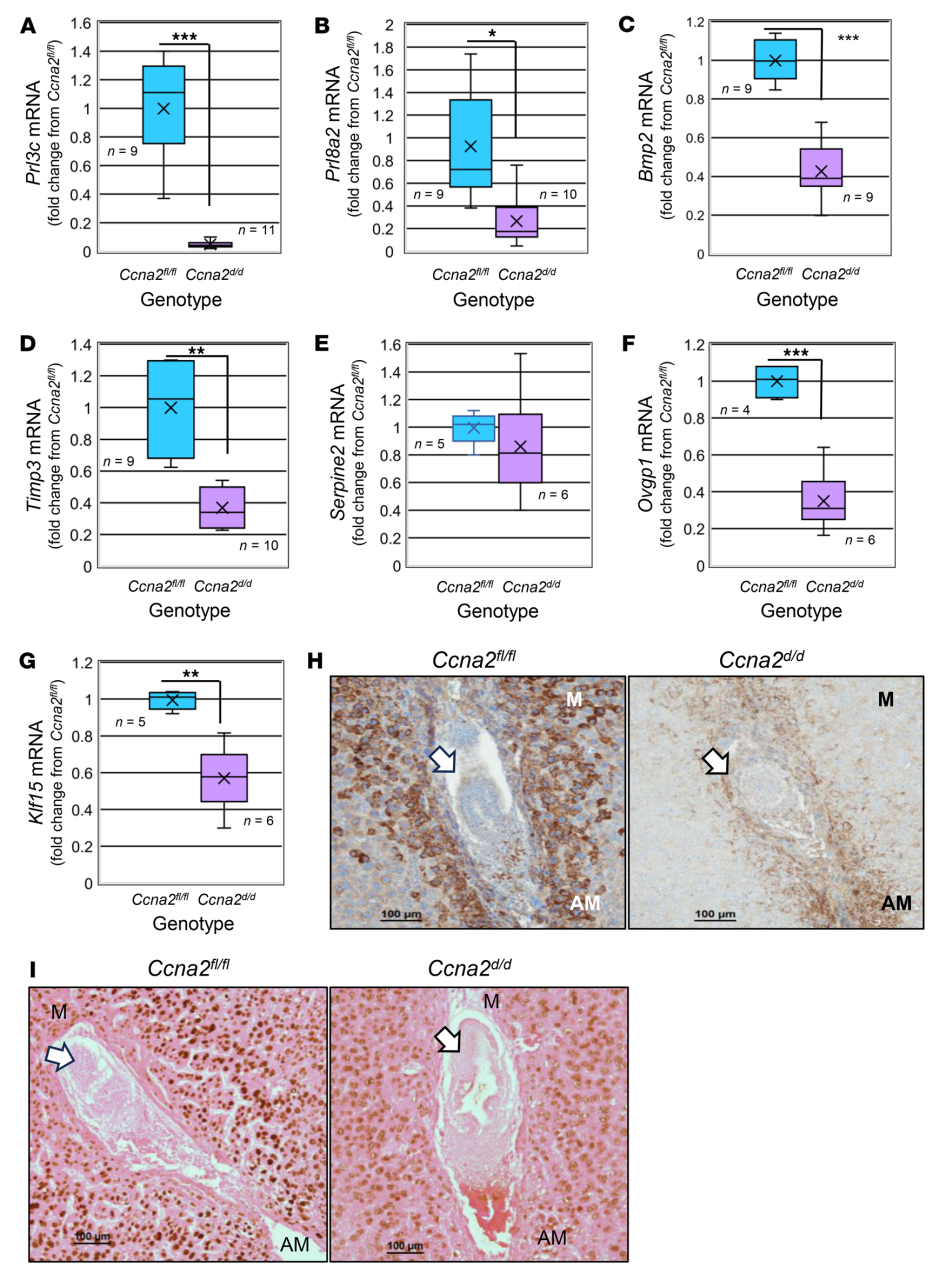
Ccna2 is essential for optimal decidualization in vivo. (**A**–**G**) qRT-PCR was performed using RNA extracted from dpc 6.5 implantation sites (*n* = 1–2/mouse) from *Ccna2^fl/fl^* and *Ccna2^d/d^* females (sample size is indicated in each bar graph/genotype) for decidualization markers (**A**) *Prl3c*, (**B**) *Prl8a2*, (**C**) *Bmp2*, (**D**) *Timp-3*, (**E**) *Serpine1*, (**F**) *Ovgp1*, and (**G**) *Klf15*. Decidualization was also evaluated in implantation sites by immunohistochemical localization of (**H**) Ptgs2 (Cox2) and (**I**) Pgr (*n* = 5 /genotype) between genotypes. Brown color indicates positive staining, and intensity is greater in *Ccna2^fl/fl^* mice compared with *Ccna2^d/d^* mice for both Ptgs2 and Pgr. M, mesometrial side; AM, antimesometrial side. Arrows indicate embryo. Scale bars: 100 μm.

To evaluate whether loss of Ccna2 prior to mating and oocyte fertilization compromises the subsequent decidualization process, we assessed markers of decidualization at dpc 6.5 in mice of both genotypes using both qRT-PCR and immunohistochemical localization methodology. Compared with *Ccna2^fl/fl^* dpc 6.5 implantation sites, expression of *Prl3c1* (Figure 6A) and *Prl8a2* (Figure 6B) was reduced in tissues from *Ccna2^d/d^* mice. Similarly, expression of the stromal cell enriched proteins *Bmp2* (Figure 6C) and *Timp3* (Figure 6D) was also reduced. However, expression of *Serpine1*, which is also stromal cell enriched and associated with successful decidualization, did not change between genotypes (Figure 6E). As *Klf15* (27–29) and *Ovgp1* (24, 33, 34) misexpression was reported to be associated with infertility, we also assessed their expression. Both *Ovgp1* (Figure 6F) and *Klf15* (Figure 6G) expression were lower in dpc 6.5 implantation sites from *Ccna2^d/d^* mice. Assessment of the implantation marker, prostaglandin-endoperoxide synthase 2/cyclooxygenase-2 (Ptgs2/Cox2; ref. 40) revealed diminished intensity of staining around the implanted embryo (Figure 6H). Expression of PGR, which is also essential for decidualization, was markedly reduced in the decidual tissue of *Ccna2^d/d^* mice as well (Figure 6I); this supports earlier observations of reduced PGR when CCNA2 expression is reduced.

### CCNA2 knockdown in vitro reduces the expression of decidualization-specific genes in t-HESC.

**Figure 7:**
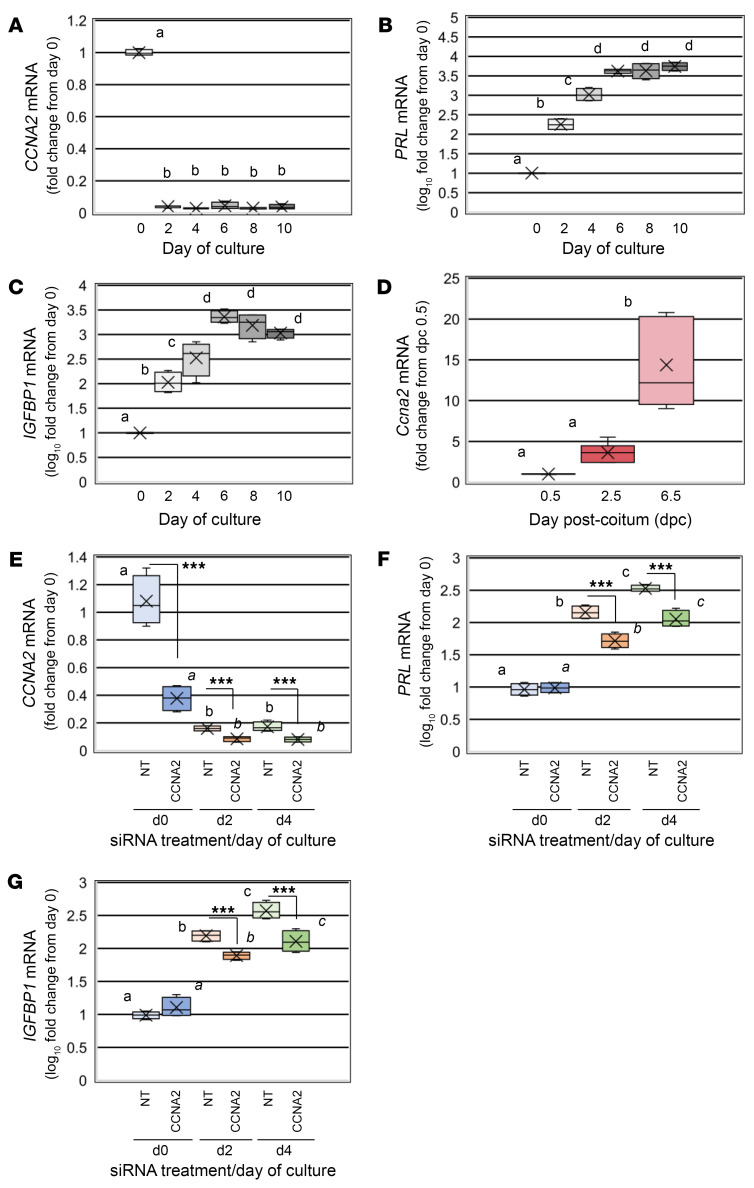
CCNA2 expression in human and mouse uterine stromal cells and uterine tissue prior to and during decidualization. (**A**–**C**) qRT-PCR assessment of (**A**) *CCNA2*, (**B**) *PRL*, and (**C**) *IGFBP1* transcript expression during in vitro decidualization in nontransfected t-HESCs. (**D**) *Ccna2* expression prior to and during late decidualization in mouse uterine tissue. Data are normally distributed and analyzed by 1-way ANOVA followed by Bonferroni’s post-hoc analysis. Different letters indicate statistical significance among the groups. In **D**, a versus b = *P* < 0.001. *P* < 0.001 for *n* =6 mice for dpc 0.5 and 2.5 and *n* = 5 mice for dpc 6.5. (**E**–**G**) qRT-PCR analysis of (**E**) *CCNA2*, (**F**) *PRL*, and (**G**) *IGFBP1* transcript expression in NT-siRNA and CCNA2-siRNA transfected t-HESC prior to (day 0), and after a decidualization stimulus. Different block letters indicate statistical significance among the means across days of culture/treatment groups for NT-siRNA-transfected cells while different letters in italics indicate statistically significant differences among the means in the CCNA2-siRNA-transfected cells using 1-way ANOVA followed by Tukey-Kramer multiple comparison tests. In **E**, a versus b = *P* < 0.001. *a* versus *b* = *P* < 0.001; in **F**, a versus b = *P* < 0.001, a versus c = *P* < 0.001, b versus c = *P* < 0.001; *a* versus *b* = *P* < 0.001, *a* versus *c* = *P* < 0.001, *b* versus *c* = *P* < 0.01; in **G**, a versus b = *P* < 0.001, a versus c = *P* < 0.001, b versus c = *P* < 0.001; *a* versus *b* = *P* < 0.001, *a* versus *c* = *P* < 0.001, *b* versus *c* = *P* < 0.05. ***P < 0.001 between transfection groups within each time point using unpaired, 2-tailed *t* tests. For Figure 7, B and C, as well as Figure 7, E and F, values are expressed as the log_10_ value of the FC from the appropriate control for each transcript. Data are from 4 separate experiments (*n* = 4 replicates with different cell passage numbers). Within each graph of the box and whisker plot, X indicates the mean values while the whisker end points indicate the highest and lowest values.

To provide further proof of principal that CCNA2 deficiency prior to a decidualization stimulus compromises the decidualization process, we utilized the well-described in vitro decidualization model using t-HESCs (39). As the pattern of CCNA2 expression during in vitro and in vivo decidualization is unknown, we first assessed transcript expression by qRT-PCR in t-HESCs and then uterine tissue from *Ccna2^fl/fl^* mice prior to (dpc 0.5, 2.5) and during late decidualization (dpc 6.5). In t-HESCs, *CCNA2* transcript levels were highest prior to induction of in vitro decidualization and rapidly declined and remained low from days 2 through 10 (Figure 7A), showing an inverse relationship with the well-known markers of decidualization prolactin (*PRL*) (Figure 7B) and insulin-like growth factor binding protein 1 (*IGFBP1*) (Figure 7C). In contrast, in *Ccna2^fl/fl^* mice, *Ccna2* expression was low at dpc 0.5 and increased at dpc 2.5 as well as at dpc 6.5 (Figure 7D). The discrepancy between the in vitro t-HESC and in vivo mouse patterns of CCNA2 expression may be in part due to lack of cell-cell and cell-matrix interactions in the former, as it is well established that endometrial glands are essential for decidualization (35–38). This postulate is currently being tested by developing 3D culture systems integrating stromal and epithelial cells.

We next set out to demonstrate that reduction of CCNA2 prior to decidualization (which occurs in our *Ccna2^d/d^* mouse model) negatively impacts the decidualization process. We transfected t-HESCs with *CCNA2* siRNA or a NT siRNA prior to decidualization. Twenty-four hours after transfection, we changed the media to decidualization media and harvested the cells at day 0 (prior to decidualization stimulus), day 2, and day 4 to assess the expression of decidualization markers *PRL* and *IGFBP1*. Knockdown of CCNA2 was confirmed on day 2 and day 4 CCNA2 siRNA–transfected cells (Figure 7E) as was the pattern of CCNA2 expression in the NT siRNA–transfected cells (Figure 7E). Expression of *PRL* (Figure 7F) and *IGFBP1* (Figure 7G) increased in cells transfected with NT siRNA on day 2 and day 4 compared with day 0. However, the expression of *PRL* (Figure 7F) and *IGFBP1* (Figure 7G) in *CCNA2*-deficient cells was lower compared with control cells on day 2 and day 4. Thus, loss of CCNA2 in both t-HESC cells and mouse uterine tissue prior to a decidualization stimulus resulted in compromised decidualization.

## Discussion

Successful pregnancy requires a suitable endometrial environment, stromal cell decidualization, and placental development, all of which are dependent upon proper steroid signaling. Cyclins (CCNs) are a family of proteins that, along with their CDKs control the progression of the cell cycle. CCNs and CDKs have been reported to exhibit noncanonical functions (11) as well as mediating E2 (12, 13) and P4 (12, 14–16) signaling. Cyclins and CDKs have been examined in normal uterine tissue of humans (41–51) and mouse (52) with most of the emphasis on CCNE and CCND family members. Little information is available for CCNA2, which is 1 of 2 members of the CCNA family (CCNA1 and CCNA2). Reports on CCNA (reports did not specify if they assessed CCNA1 or CCNA2 expression) have primarily focused on endometrial carcinoma tissue, but when normal endometrium was assessed, CCNA expression was low to absent (49–51) in these tissues with any expression localized to endometrial epithelium. CCNA1 is restricted to the germ line (53, 54), while CCNA2 is ubiquitously expressed in all proliferating cells and is upregulated in a variety of cancers (55) including endometrial cancer (49–51). In the current study, low/deleted levels of CCNA2 in both human and mouse tissues, respectively, were not associated with major differences in cell proliferation, which may be attributed to potential compensation by other cyclins, such as CCNE, which was reported in the Ccna2-null mice (56).

In addition to regulating cell proliferation, CCNA2 was also demonstrated to mediate estrogen (E2; refs. 12, 13) and P4 (12, 14–16) receptor signaling. CCNA2 may modulate steroid action and act as a coregulator. CCNA2 expression was significantly (P < 0.01) lower in biopsies from subjects who failed to achieve pregnancy, and this was associated with lower expression of ERα and PGR expression primarily within the stromal cells. Using t-HESC cells, we demonstrated that knockdown of CCNA2 resulted in reduced E2 induction of *PGR* expression. In *Ccna2^d/d^* mice, E2 induction of whole uterine tissue *Pgr* expression was reduced in ovariectomized mice and reduced levels of Pgr expression were also observed in the *Ccna2^d/d^* mice at dpc 0.5 and dpc 6.5 with the former associated with lower levels of phosphorylated (ser104) Erα. In contrast to the human endometrial tissue, Erα expression did not differ between genotypes at dpc 0.5. Collectively, our human and mouse data are interpreted to suggest that CCNA2 impacts stromal cell P4 receptor expression, which may in turn contribute to the impaired fertility in both human and mouse.

Perhaps the most dramatic effect of Ccna2 deficiency was the substantial reduction in E2 induction of uterine wet weight in ovariectomized *Ccna2^d/d^* mice. Analysis of the 0-hour mouse uterine tissue revealed that uteri of the *Ccna2^d/d^* mice were approximately one-third the weight of those of control mice. While E2 induced increases in uterine wet weight in mice of both genotypes, uteri of *Ccna2^d/d^* mice never reached similar uterine wet weights at either 6 hours or 24 hours after E2 treatment. Estrogen regulation of some target genes was less affected. More specifically, analysis of differential gene expression during the early and late E2 responses revealed that Erα-responsive genes were not affected globally by the deletion of *Ccna2* in the uterus. We did not detect differences in the gene expression of *Cstb*, *Serpine1*, and *Timp1* between control and *Ccna2^d/d^* mice by qRT-PCR; however, regulation of *Fgf10* (57) and *Pgr* were compromised in *Ccna2^d/d^* mice and exhibited lower levels at 24 hours after E2 administration compared with *Ccna2^fl/fl^* mice.

In the current study, we wished to emphasize focus on endometrial tissue specimens obtained prior to the window of implantation, specifically during the stage of endometrial tissue proliferation. The majority of studies (58–63) have focused on the endometrial environment during the secretory stage of the menstrual cycle with emphasis on the window of embryo implantation (which typically begins on day 19/20 of an idealized 28-day menstrual cycle and lasts approximately 4 to 5 days; ref. 58). However, it is becoming apparent that events that occur during the menstrual and proliferative stages of the menstrual cycle when regeneration and proliferation of the endometrium occur may also play a role in establishing an optimal endometrial environment for successful term pregnancy. Brosens and colleagues proposed a role for menstruation in preconditioning of the uterus for successful pregnancy (9), while Bromer and associates (10) postulated that diseases associated with infertility may manifest a developmental defect during the proliferative phase of the menstrual cycle which may contribute to difficulty in establishing pregnancy. However, until recently, few mechanistic studies have been conducted to support or refute these postulates. A recent study by Lv and coworkers (61) evaluated the endometrial niche of human thin endometrium in specimens obtained during the LP phase of the subject’s normal menstrual cycle using single-cell RNA-Seq. In subjects with thin endometrium (defined as an endometrial thickness < 7 mm at midluteal phase or histories of embryo transfer cancellations of IVF procedures due to thin endometrium), the investigators reported inhibition of stromal cell proliferation in thin endometrium specimens which was associated with impaired epidermal growth factor (EGF), pleiotrophin (PTN), and TWEAK signaling pathways and overactivation of the SEMA3B pathway. Further, the defective growth of the thin endometrium/decreased proliferation was associated with increased cellular senescence and collagen deposition. One of the most striking observations in our study with respect to the *Ccna2^d/d^* null mice was the hypoplastic uteri, which exhibited a thin endometrium compared with *Ccna2^fl/fl^* mice in both the naturally mated dpc 0.5 groups as well as the E2-treated ovariectomized mice. However, in the study by Lv and associates (59), thin endometrium was associated with increased expression of markers of cell senescence (*p16*, *p21*) and collagen deposition (*Col4a1*). In our study, we did not detect major differences in expression of these genes in *Ccna2^d/d^* mice, although the genes for p16 (*Cdkn2a*) and p21 (*Cdkn1a*) exhibited higher expression levels (approximately 3- and 2-fold, respectively, but did not meet our inclusion criteria for further assessment) compared with *Ccna2^fl/fl^* mice, while *Col4a1* was not differentially expressed between genotypes. Potential explanations may be related to species differences or the fact that in the study by Lv and colleagues (59), “thin” endometrium was obtained from patients who had multiple dilation and curettage (D&C) procedures while the controls did not undergo such procedures, which may confound outcomes. With respect to our human specimens in our study, only 1 subject had a prior D&C procedure, but this subject achieved pregnancy on a subsequent frozen embryo transfer cycle.

In assessing infertility (failed embryo implantation, early pregnancy loss), most focus has been on the window of implantation. Recent evidence suggests a suitable implantation environment may first be established prior to embryo implantation during the proliferative stage of the menstrual cycle if not earlier. Our animal model in which *Ccna2* is deleted from the uterine tissue prior to puberty would support this notion.

Similar to the observation of increased collagen/ECM deposition in thin endometrium and infertility in humans (59), uteri from *Ccna2^d/d^* mice at dpc 0.5 exhibited alterations in expression of genes associated with the matrisome. Of these, we confirmed increased expression of tenascin C (*Tnc*) and *Ctgf* (also known as *Ccn2*). Both *Tnc* (46, 47) and *Ctgf* (48, 49) are steroid-regulated genes and may potentially play a role in RPL/altered decidualization. Of the downregulated genes, 2 of the most decreased genes were epithelial-specific genes *Klf15* (24–26) and *Ovgp1* (21, 30, 31), and like *Tnc* and *Ctgf*, both are steroid-regulated and have been associated with embryo implantation and decidualization as well. Currently, how disruption of Ccna2 expression may lead to increased expression of *Tnc* and *Ctgf* is unknown, as is the mechanism for reduced expression of *Klf15* and *Ovgp1*. Considering that both *Klf15* and *Ovgp1* are epithelial-specific genes, the observation that the total number of endometrial glands are reduced in *Ccna2^d/d^* uteri may offer some explanation for their reduced expression. The necessity for endometrial glands (and their secretions) for successful pregnancy is clearly established (35–38). Despite a reduction in the number of endometrial glands, *Ccna2^d/d^* females were still capable of achieving early pregnancy, but were incapable of supporting pregnancy to term. Whether the reduced number of glands in these mice contributed to later loss of pregnancy is unknown.

Perhaps the most substantial finding from the current study was the observation that endometrial tissue from women who fail to achieve pregnancy after an assisted reproductive technology–assisted (ART-assisted) cycle express very low levels of CCNA2 and that this reduction was independent of diagnosis of infertility cause. While we are aware that there may be cycle-to-cycle variation in CCNA2 in endometrial biopsies (62), our own unpublished Igenomix data would suggest cycle-to-cycle variation may be minimal. Thus, CCNA2 may play an important role in fertility and may be part of the explanation for unexplained infertility; however, larger sample sizes will be required to further support this possibility. The mechanism by which CCNA2 may affect decidualization prior to a decidualization stimulus remains uncertain, but both our human in vitro and mouse in vivo data suggest that reduced levels of the P4 receptor may play a role. Of the known transcription factors with documented roles in the decidualization process (63), only FosB proto-oncogene, AP-1 transcription factor subunit (Fosb) was differentially expressed in the dpc 0.5 uteri of *Ccna2^d/d^* mice exhibiting a 2.1-fold increase in expression compared with control counterparts. Fosb is one of the 4 FOS family members that can dimerize with proteins of the JUN family to form the AP-1 transcription complex (64), which in turn induces expression of key decidualization factors (65). How increased Fosb may impede decidualization is unknown, but it may be associated with altered cell dynamics. Overexpression of FOSB decreased the transformation phenotype of gastric cancer cell lines in vitro (66), and Fosb expression was linked to elevated levels of cyclooxygenase-2 (COX2) in colon adenocarcinoma HCA-7 cells (67). As Cox2 expression was markedly lower in implantation sites from *Ccna2^d/d^* mice and given the fact that Cox2 plays a vital role in decidualization (68), it is tempting to speculate that higher FosB expression in these mice may be a compensatory effort to restore Cox2 levels.

In summary, we report that CCNA2 levels are substantially lower in endometrial tissue obtained during controlled ovarian stimulation protocols from subjects who fail to achieve pregnancy compared with those who do. Using a conditional Ccna2-knockout mouse model in which Ccna2 is deleted from uterine tissue, we further demonstrate that loss of this cyclin results in impaired decidualization, altered placenta development, and inability to maintain term pregnancy. As low (human) or deleted (mouse) expression of CCNA2 occurs in both species prior to ovulation, studies are ongoing to explore at what point during uterine development CCNA2 expression is disrupted and identify the mediators and mechanisms in humans.

## Methods

### Sex as a biological variable.

As this study focused on early pregnancy and only females can become pregnant, males were excluded from this study.

### IHC and H-score.

To assess the expression and localization pattern of CCNA2, Ki67, ERα, and PR in the endometrium, IHC was performed following the protocol of the reagent supplied (Vector Laboratories Inc.) using commercially available antibodies (Supplemental Table 3). First, the slides containing the tissue section were deparaffinized with xylene, then rehydrated with a series of 100%, 95%, and 75% ethanol. After a washing step, slides were incubated in antigen-unmasking solution at 95°C (H-3300, Vector Laboratories Inc.) for antigen retrieval. After that, slides were incubated in 1× PBS on ice, then quenched with 3% hydrogen peroxide. The VECTASTAIN ABC Kit (Vector Laboratories, PK-6101) was used for the IHC assay. Slides were incubated with diluted normal serum provided by the kit, then primary antibody for 1 hour at room temperature. Then slides were washed and incubated with the biotinylated secondary antibody provided by the kit. After that, avidin/biotinylated enzyme complex (ABC) was added. After that, ImmPACT DAB peroxidase (HRP) substrate (SK-4100, Vector Laboratories, Inc.) was used to develop a brown color. Then slides were counterstained with hematoxylin and dehydrated with graded ethanol and xylene. Then slides were mounted and coverslipped with VectaMount (H-5000, Vector Laboratories, Inc.). The expression level and localization of each target was quantified using the H-score system. Each slide was blindly evaluated at ×20 field by 2 reviewers. The intensity of staining was scored individually as 0 = no staining, 1 = minimal staining, 2 = moderate, 3 = strong of each cell type (stroma and glandular epithelium). The average intensity was calculated using the following equation: (H-score for each cell type = average intensity x% of cell type × 100%), as previously reported by our group (69).

### Stranded total RNA library RNA-Seq.

Stranded total RNA-Seq was performed using the Illumina NovaSeq 6000 Sequencing System at the University of Kansas Medical Center – Genomics Core. Quality control of the total RNA submissions was completed using the Agilent TapeStation 4200 using the RNA ScreenTape Assay Kit (Agilent Technologies, 5067-5576). Total RNA (500 ng) was used to initiate the Illumina Stranded Total RNA Prep Ligation with Ribo-Zero Plus (Illumina, 20040525) library preparation protocol. The total RNA fraction underwent a probe-based ribosomal reduction using Ribo-Zero Plus probes which are hybridized to rRNA targets with subsequent enzymatic digestion to remove the overabundant rRNA from the purified total RNA. The ribosomal depleted RNA fractions were sized by fragmentation, reverse transcribed into cDNA, end repaired, and ligated with the appropriate indexed adaptors using the IDT for Illumina RNA UD unique dual indexes (UDI) (Illumina 20040553) to yield strand-specific RNA-Seq libraries.

Following Agilent TapeStation D1000 QC validation of the library preparation, the final library quantification by qPCR was performed using the Roche Lightcycler96 with FastStart Essential DNA Green Master (Roche, 06402712001) and KAPA Library Quant (Illumina) DNA Standards 1-6 (KAPA Biosystems KK4903). The RNA-Seq libraries were normalized to a 2 nM concentration and pooled for multiplexed sequencing on the NovaSeq 6000.

Pooled libraries were denatured with 0.2N NaOH (0.04N final concentration) and neutralized with 400 mM Tris-HCl pH 8.0. The pooled libraries were diluted to 380 pM prior to onboard clonal clustering of the patterned flow cell using the NovaSeq 6000 S1 Reagent Kit, version 1.5 (200 cycle) (Illumina, 20028318). A 2 × 101 cycle sequencing profile with dual index reads was completed using the following sequence profile: read 1 – 101 cycles × index read 1 – 10 cycles × index read 2 – 10 cycles × read 2 – 101 cycles. Following collection, sequence data were converted from.cl file format to fastq file format using bcl2fastq software and demultiplexed into individual sequences for data distribution using a secure FTP site or Illumina BaseSpace for further downstream analysis.

### Gene ontology analyses.

Gene ontology analyses were conducted on DEGs whose expression was greater than 1.5-fold (increased or decreased) between genotypes with *P* < 0.05 and FDRs < 0.1. DEGs were subjected to enrichment analysis using the Metascape platform (70).

### P4 and E2 ELISA.

E2 serum levels were quantitated using a rat/mouse ELISA kit (ES180S-100; Calbiotech), while P4 serum levels were quantitated using a rat/mouse ELISA kit (IB79183, Immuno-Biological Laboratories, Inc.; IBL-America). Briefly, mice were sacrificed by decapitation at the indicated time points, blood collected, and serum separated. Serum samples were undiluted (E2) or diluted 1:10 (P4; 10 μL serum + 90 μL diluent) and subjected to the assays following the provided protocols. After adding the stop solution to each well, the absorbance at 450 was determined for each sample using a microplate reader (FlexStation3 Multi-Mode Microplate Reader, Molecular Devices). The concentration for each sample was calculated using the standard curve of the calibrators provided as follows: 17β-estradiol (E2), standard range: 3–300 pg/mL, sensitivity: 3 pg/mL, intra-assay coefficient of variation (CV) = 3.1%, and inter-assay CV = 9.9%; P4 ELISA, standard range: 0.4 –100 ng/mL, sensitivity: 0.156 ng/mL, intra-assay CV = 7.9%, and inter-assay CV = 9.6%.

### Prolactin ELISA.

To measure prolactin concentration in mouse sera, blood was collected at the indicated time points as described above and serum prolactin (diluted 1:10) was quantitated using mouse prolactin ELISA PicoKine kits (Boster Biological Technology, following the provided protocol). After adding the stop solution to each well, the absorbance at 450 was determined for each sample using a microplate reader (FlexStation). The concentration for each sample was calculated using the standard curve of the calibrators provided as follows: standard range: 15.6–1,000 pg/mL, sensitivity: <10 pg/mL; intra-assay CV = 4.6% and inter-assay CV = 5.8%.

### Cell culture.

Immortalized human endometrial stromal cells (t-HESC; 39) were obtained from ATCC (CRL-4003) and were maintained with DMEM-F12 (Fisher/Corning) media supplemented with 10% FBS (non-charcoal stripped FBS), 1% penicillin/streptomycin, and 2 μl/ml normocin. For cell transfection, t-HESCs were seeded in 6-well plates at a density of 350,000 cells/well and cultured in DMEM-F12 with 2% charcoal stripped FBS and 1% penicillin/streptomycin. Lipofectamine 2000 was used to prepare the *CCNA2* or NT siRNA solution. t-HESCs were transfected with *CCNA2* siRNA or NT siRNA prior to short-term E2 decidualization induction. Twenty-four hours later, media was changed to either short-term E2 (10 nM) or vehicle (100% ethanol at 0.01% v/v) for 6 hours to assess *PGR* expression or to decidualization media which contained 5% horse serum, 1 μM medroxyprogesterone acetate, 10 nM E2, and 0.5 mM 8-bromoadenosine 3′,5′-cyclic monophosphate with media changes every 48 hours. t-HESCs were harvested at day 0, day 2, and day 4 to assess the expression of decidualization markers *PRL* and *IGFBP1*.

### RNA isolation and qRT-PCR assessment.

qRT-PCR was performed as previously described (17, 69). Briefly, total RNA was isolated from tissue or cells using Tri-Reagent (Sigma Chemical Co.) according to recommendations of the manufacturer. Total RNA (1 μg in 20μL) was reverse transcribed using reverse transcription (RT) kits (Applied Biosystems) following the manufacturer’s protocol. Primers (Supplemental Table 4) were designed for human and mouse target transcripts using Primer-Blast and synthesized by Integrated DNA Technology (IDT). Resulting material was then used for independent qRT-PCR, which was carried out on an Applied Biosystems HT7900 Sequence Detector. To account for differences in starting material, human 18S rRNA primers (4333760; Thermo Fisher Scientific) were used, while for mouse tissues, ribosomal protein L13 primers (*mRpl13*; Supplemental Table 4) were used. All samples were run in triplicate and the average value used in subsequent calculations. The 2ΔΔ Ct method was used to calculate the fold-change values among samples as previously described by our group (17, 69).

### Human and mouse model information and statistical analysis.

Experimental details for human subjects, generation, genotyping, and fertility assessment of the Ccna2-deficient mice as well as statistical analysis are described in Supplemental Methods.

### Study approval.

Studies utilizing human tissues were approved by the Institutional Review Board at the University of Kansas Medical Center (HSC140116). Written, informed consent was obtained from all study subjects prior to participation. Biopsies were obtained at the University of Kansas Health System Center for Advanced Reproductive Medicine by either a reproductive endocrinologist or a registered nurse. For animal studies, all experiments and procedures were approved by the IACUC of the University of Kansas Medical Center in accordance with the *NIH Guide for the Care and Use of Laboratory Animals* (National Academies Press, 2011) (protocol #22-11-280).

### Data availability.

RNA-Seq data can be accessed at the NCBI’s Gene Expression Omnibus database (GEO GSE272413). Values for all data points in graphs are reported in the Supporting Data Values file.

## Author contributions

The study was designed by WBN, FA, KS, and CM. Experiments were conducted by WBN, FA, KS, and AG. CM, ML, and KH identified subjects who satisfied the inclusion/exclusion criteria, obtained consent, and obtained biopsies. WBN, FA, KS, and CM wrote the draft manuscript. WBN, FA, and SG analyzed data. All authors read and approved the final manuscript.

## Supplementary Material

Supplemental data

Supporting data values

## Figures and Tables

**Table 1:**
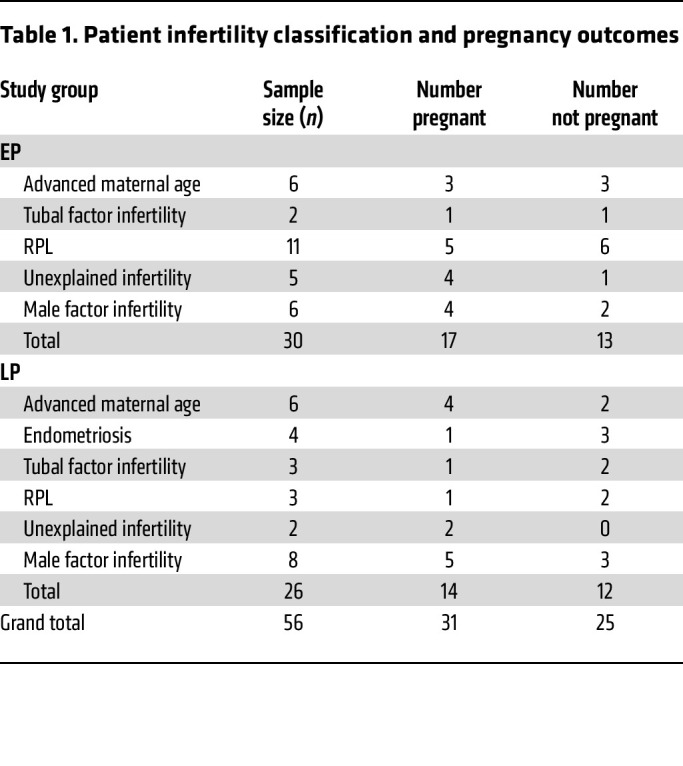
Patient infertility classification and pregnancy outcomes

## Version 1 09/12/2024

In-Press Preview

## Version 2 11/15/2024

Electronic publication
