# Supplementary material for: Uterine cyclin A2–deficient mice as a model of female early pregnancy loss
Source: J Clin Invest. 2024 Sep 12;134(22):e163796. doi: 10.1172/JCI163796 (PMC11563677; doi:10.1172/JCI163796)
Supplement: Supplemental data [file jci-134-163796-s184.pdf]

Supplemental materials

Supplemental methods

Human sample procurement.

This study was approved by the Institutional Review Board at the University of Kansas Medical Center. Our cohort study consists of women undergoing in vitro fertilization (IVF) cycles for infertility treatment. The patients were diagnosed with one of the following conditions: advanced maternal age, endometriosis, male factor only, tubal factor infertility, recurrent pregnancy loss, or unexplained infertility (Table 1). Endometrial samples were obtained from a non-conception cycle and pregnancy outcomes were reported as positive/negative pregnancy based upon beta-hCG levels. All human endometrial samples obtained in follicular phase (menstrual cycle day 3-6; n = 30) were on no exogenous hormones. All day 11 to 13 (late proliferative; n = 26) endometrial samples were obtained in the peri-ovulatory phase of cycle obtained at time of oocyte retrieval. Stimulation protocol for study participants included daily subcutaneous injections of recombinant FSH and human menopausal gonadotropins for 9 to 11 days with doses ranging from 75-450 IU. Doses were set by the physician (CM, ML or KH) using antral follicle count, age, and anti-mullerian hormone levels. Ovulatory suppression was gonadotropin releasing hormone antagonist started with 13-14 mm follicle for over 90% of study participants. Three participants had micro-dose leuprolide acetate subcutaneous injection twice daily to prevent ovulation. Trigger to mature oocytes occurred 35-36 hours before retrieval and was either leuprolide acetate 4mg or human chorionic gonadotropin 10,000 U per physician preference. Six participants had

additional stimulation hormones including clomiphene citrate, letrozole, testosterone patch priming to boost ovarian response. Variations in these stimulation and triggering protocols were not associated with differences in pregnancy outcomes or CCNA2 expression. Endometrial biopsies (50 – 100 mg of tissue) were obtained using a pipelle instrument and placed directly into 10% neutral-buffered formalin for transport to the research laboratory. Endometrial tissue was prepared for paraffin embedding and serial sectioning. Resulting tissue sections (5 μm sections) were mounted onto glass slides for immunohistochemistry.

Animals. Floxed *Ccna2* mice on a C57BL/6 background (1) were crossed with Pgr^{cre} mice (C57BL/6 background) to generate conditional knockout mice and all lines were maintained on a pure C57BL/6 background. The cre recombinase mediates *Ccna2* deletion in *Pgr* expressing tissues ($Ccna2^{fl/fl}$; $Pgr^{cre/+}$, referred here in as $Ccna2^{d/d}$). Floxed *Ccna2* ($Ccna2^{fl/fl}$) mice were generously provided by Dr. Peter Sicinski at Dana-Farber Cancer Institute, Boston, MA, while Pgr^{cre} mice were generously provided by Dr. John P. Lydon at Baylor College of Medicine, Houston, TX. Control ($Ccna2^{fl/fl}$) and $Ccna2^{d/d}$ female mice were sacrificed at the indicated ages. At the indicated time points of sacrifice, uterine morphology was grossly evaluated and uterine wet weight determined. Measurement of body weight and uterine wet weight are taken prior to tissue processing for endpoint analysis. The uterus was divided and preserved in RNALater solution or 10% Neutral Buffered Formalin (NBF) until processed for RNA isolation or immunohistochemistry as described below.

Genotyping. To obtain DNA, ear punch biopsies were obtained from mice between 14 and 17 post-natal days of age. DNA was extracted and PCR reactions performed to identify the genotype of the mouse. DNA extraction was done using REDExtract-N-Amp Tissue PCR kit (XNAT, Sigma). For the PCR reaction, REDExtract-N-Amp PCR ReadyMix (R4775, Sigma) was used. CCNA2 fl/fl primers: CCNA2 loxP1 forward 5' - CGCAGCAGAAGCTCAAGACTCGAC- 3', CCNA2 loxP1 reverse 5' - TCTACATCCTAATGCAATGCCTGG- 3', and CCNA2 deleted 5' - CACTCACACACTTAGTGTCTCTGG- 3'. PR-Cre primers: PR-Cre 1 5' - ATGTTTAGCTGGCCCAAATG- 3', PR-Cre 2 5' -TATACCGATCTCCCTGGACG- 3', and PR-WT 5' - CCCAAAGAGACACCAGGAAG- 3'.

Fertility assessment. Control (*Ccna2*$^{fl/fl}$) and *Ccna2*$^{d/d}$ female mice were mated with wild-type (C57BL/6) males of proven fertility. Females were checked daily for presence of copulatory plugs to confirm mating, with the detection of a copulatory plug considered day post coitum (dpc) 0.5. Plugged females were housed separately and allowed to deliver to term. On term date, the number of pups born were recorded. In the case of no pups born, *Ccna2*$^{d/d}$ were sacrificed to assess fetal resorption.

Ovariectomy and hormonal treatment. *Ccna2*$^{fl/fl}$ and *Ccna2*$^{d/d}$ female mice were ovariectomized following standard procedures at 2 – 3 months of age, rested 14 days and then treated with estradiol (E2; 10 µg/kg BW). Mice were sacrificed at the indicated time points, uterine tissue wet weight determined and uterine tissues were processed

for either histological assessment or RNA extraction and subsequent qRT-PCR analysis.

Evaluation of pregnancy. *Ccna2$^{fl/fl}$* and *Ccna2$^{d/d}$* female mice were mated with wild-type (C57BL/6) males of proven fertility as described under "Fertility assessment." Plugged females were housed separately and sacrificed at dpc 0.5, 2.5, 6.5, 10.5, or 13.5. On dpc 0.5, the oocytes were retrieved from the oviduct by gently tearing open the oviduct with fine forceps and counted. Blood samples were collected immediately after decapitation from all mice at all dpc timepoints. Serum samples was obtained by allowing blood samples to clot at room temperature for 30 – 45 minutes followed by centrifugation at 3800 rpm at 22 C°. The supernatant (serum) was removed into clean 1.5 mL microfuge tubes and stored at -80 C° until assessed for estradiol, progesterone and/or prolactin content. Uterine samples were obtained immediately after blood collection for RNA assessment and histology.

Assessment of endometrial gland density. To assess the number of endometrial glands, tissue sections from mice of both genotypes (at dpc 0.5) were subjected to IHC localization as described under "Immunohistochemistry (IHC) and H-score" for cytokeratin-19 (KRT19) to visualize glands. The number of glands per section were then manually counted using a Nikon microscope for 2 separate sections in one uterine horn for each mouse (*n* = 4 mice/genotype). The average number of glands/cross section was then calculated by taking the average of the 2 sections.

Assessment of phosphor-ser104 ERα and phosphor-ser106 ERα at dpc 0.5. To assess phosphorylation of ERα at serine residues 104 and 106, tissue sections from mice of both genotypes (at dpc 0.5) were subjected to IHC localization as described under "Immunohistochemistry (IHC) and H-score." The number of mice per genotype assessed is indicated in the respective figure legend.

Assessment of decidualization and placentation. Decidualization was assessed in dpc 6.5 and placentation in dpc 10.5 uterine sections from mice of both genotypes. Whole implantation sites were serially sectioned and we analyzed sections from the middle of the conceptus for each mouse. Decidualization was evaluated in dpc 6.5 tissue sections by immunohistochemical localization of prostaglandin-endoperoxide synthase 2/Cyclooxygenase-2 (Ptgs2/Cox2) while placental development was visualized by localization of cytokeratin9 (Krt9) using the H-score system. The number of mice per genotype per time point assessed is indicated in the respective figure legend.

Statistics. All data were first assessed for normal (Gaussian) distribution using the Kolmogorov-Smirnov test . Data which displayed normalcy of distribution were analyzed by one-way analysis of variance (ANOVA) within genotypes across treatments, while comparisons within time points/treatments between genotypes were made using two-tailed unpaired t-tests. Post-hoc analysis planned comparisons were then made using Bonferroni post-hoc analysis. Data which failed to display normality of distribution were analyzed by non-parametric tests (Mann-Whitney t-tests or Kruskal-Wallis test/one-way ANOVA on ranks) followed by post-hoc analysis using Dunn's multiple comparison tests

with planned comparisons. Data are displayed as box and whisker plots unless otherwise specified. All analysis was conducted using GraphPad Prism6 (GraphPad Software, La Jolla, CA) and significance was set at $P < 0.05$.

References

Kalaszczynska-I, et al. Cyclin A – Redundant in fibroblasts, essential in hematopoietic and embryonal stem cells. *Cell.* 2009;138(2):352 – 365.

Supplemental figures and tables

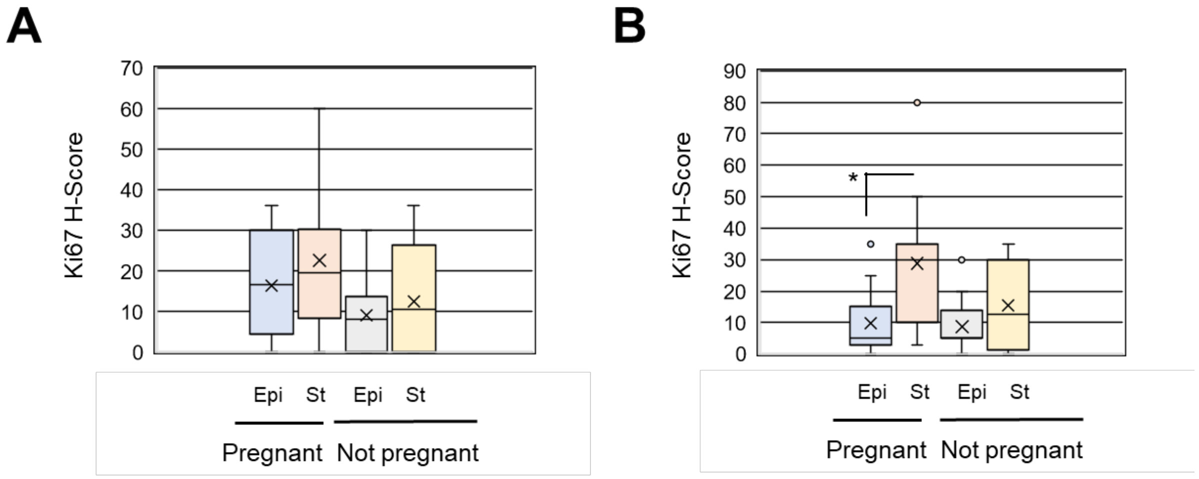

Supplemental Figure 1. Ki67 localization and expression in early and late proliferative endometrial biopsies from cisgender women who achieved or failed to achieve ART-assisted pregnancy. H-scores were calculated for Ki67 expression in endometrial biopsies during (**A**) early proliferative and late (**B**) proliferative stages of ovarian stimulation cycles. Data presented in Box and Whisker plots represent H-scores of endometrial samples in epithelial (Epi) and stromal (St) cells who either achieved a chemical pregnancy (Pregnant group) or did not (Not Pregnant group). * indicates statistical significance ($P < 0.05$) between the epithelium and stroma within Pregnant group. Within each graph of the box and whisker plot, X indicates the mean values while the whisker endpoints indicate the highest and lowest values, circles (when displayed) represent outliers which are defined as more than 1.5 times the interquartile range away from the box. H-score data were analyzed by one-way ANOVA followed by Bonferroni multiple planned comparisons within cycle stage among the four groups with P values indicated for sample sizes of $n = 14$ Pregnant and $n = 12$ Not pregnant in (A) and $n = 14$ Pregnant and $n = 12$ Not pregnant in (B).

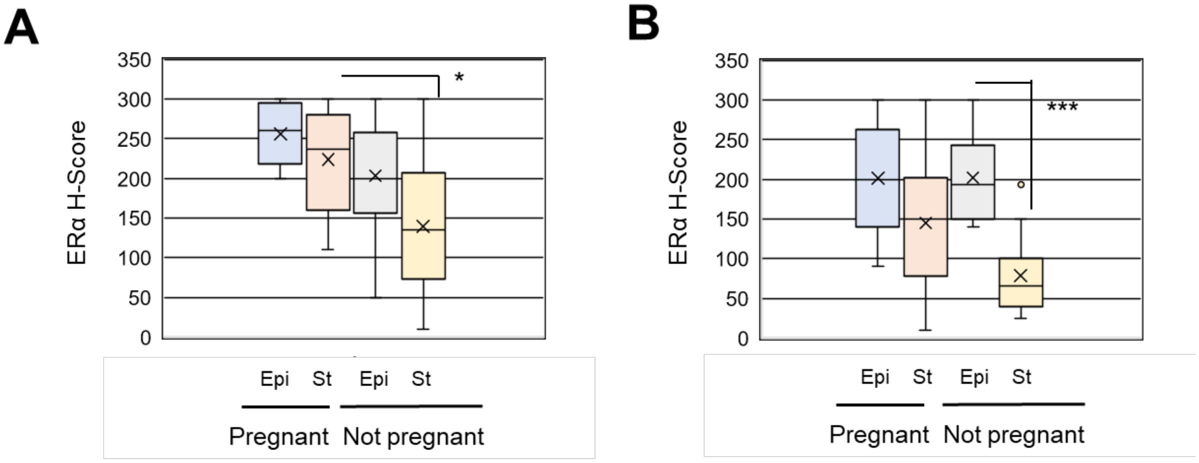

Supplemental Figure 2. Estrogen receptor-alpha localization and expression in early and late proliferative endometrial biopsies from cisgender women who achieved or failed to achieve ART-assisted pregnancy. H-scores were calculated for estrogen receptor-alpha expression in endometrial biopsies during (**A**) early proliferative and late (**B**) proliferative stages of ovarian stimulation cycles. Data presented in Box and Whisker plots represent H-scores of endometrial samples in epithelial (Epi) and stromal (St) cells who either achieved a chemical pregnancy (Pregnant group) or did not (Not Pregnant group). * ($P < 0.05$) and ** ($P < 0.001$) indicate statistical significance between stromal expression in Pregnant and Not pregnant groups (B) and between epithelial and stromal cells within the Not pregnant group (B), respectively. Within each graph of the box and whisker plot, X indicates the mean values while the whisker endpoints indicate the highest and lowest values, circles (when displayed) represent outliers which are defined as more than 1.5 times the interquartile range away from the box. H-score data were analyzed by one-way ANOVA followed by Bonferroni multiple planned comparisons within cycle stage among the four groups with P values indicated for sample sizes of n = 14 Pregnant and n = 12 Not pregnant in (A) and n = 14 Pregnant and n = 12 Not pregnant in (B).

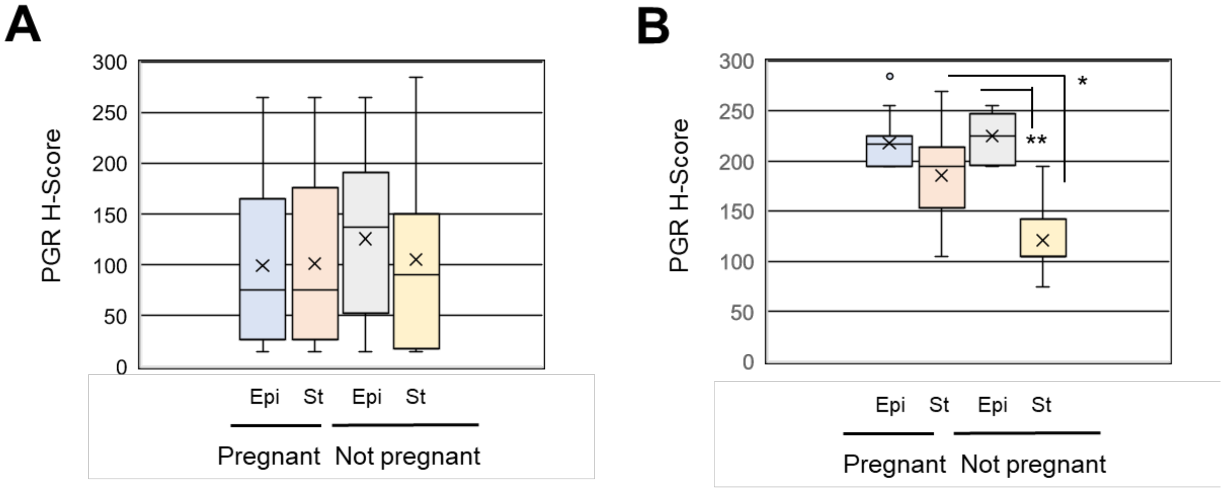

Supplemental Figure 3. Progesterone receptor localization and expression in early and late proliferative endometrial biopsies from cisgender women who achieved or failed to achieve ART-assisted pregnancy. H-scores were calculated for progesterone receptor expression in endometrial biopsies during (**A**) early proliferative and late (**B**) proliferative stages of ovarian stimulation cycles. Data presented in Box and Whisker plots represent H-scores of endometrial samples in epithelial (Epi) and stromal (St) cells who either achieved a chemical pregnancy (Pregnant group) or did not (Not Pregnant group). * ($P < 0.05$) and ** ($P < 0.001$) indicate statistical significance between stromal expression in Pregnant (A) versus Not pregnant (B) groups and between epithelial and stromal cells within the Not pregnant group (B), respectively. Within each graph of the box and whisker plot, X indicates the mean values while the whisker endpoints indicate the highest and lowest values, circles (when displayed) represent outliers which are defined as more than 1.5 times the interquartile range away from the box. H-score data were not normally distributed and were analyzed using non-parametric one-way ANOVA (Kruskal-Wallis test) followed by Dunn's multiple planned comparisons within cycle stage among the four groups with P values indicated for sample sizes of n = 14 Pregnant and n = 12 Not pregnant in (A) and n = 14 Pregnant and n = 12 Not pregnant in (B).

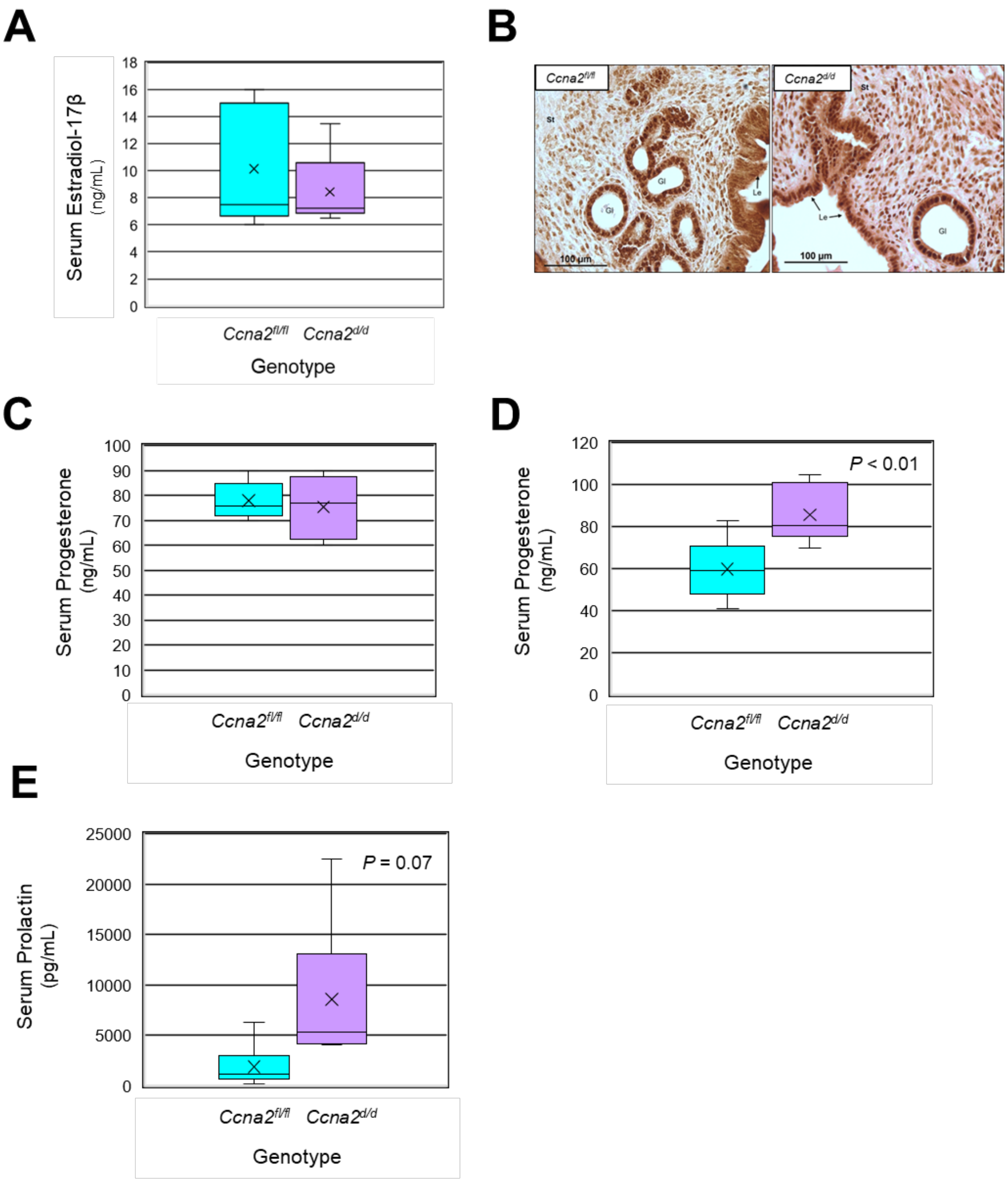

Supplemental Figure 4. Serum hormones of pregnancy and estrogen receptor-alpha expression in *Ccna2$^{fl/fl}$* and *Ccna2$^{d/d}$* female mice. Trunk blood was collected and (**A**) serum estradiol-17β levels were quantitated by ELISA (*n* = 6/genotype) while (**B**) uterine expression of estrogen receptor-alpha (Erα) was localized in uterine tissues by immunohistochemistry at dpc 0.5. Tissue sections were counterstained with eosin (*n* = 6/genotype). Brown nuclear staining represents localization of receptor expression. Gl = glandular epithelium, Le = luminal epithelium, St = stroma; scare bar = 100 µm. (**C**) Serum progesterone levels at dpc 6.5 of pregnancy (*n* = 5/genotype) were not significantly different between genotypes ($P > 0.05$). (**D**) Serum progesterone (*n* = 6/genotype) and (**E**) prolactin (*n* = 6/genotype) levels at dpc 10.5 were significantly ($P < 0.01$) higher in *Ccna2$^{d/d}$* mice compared to *Ccna2$^{fl/fl}$* controls. Data are displayed as Box and Whisker plots and were analyzed by unpaired t-tests between genotypes. Within each graph of the box and whisker plot, X indicates the mean values while the whisker end points indicate the highest and lowest values and *P*-values are provided for statistically significant differences with $P < 0.01$.

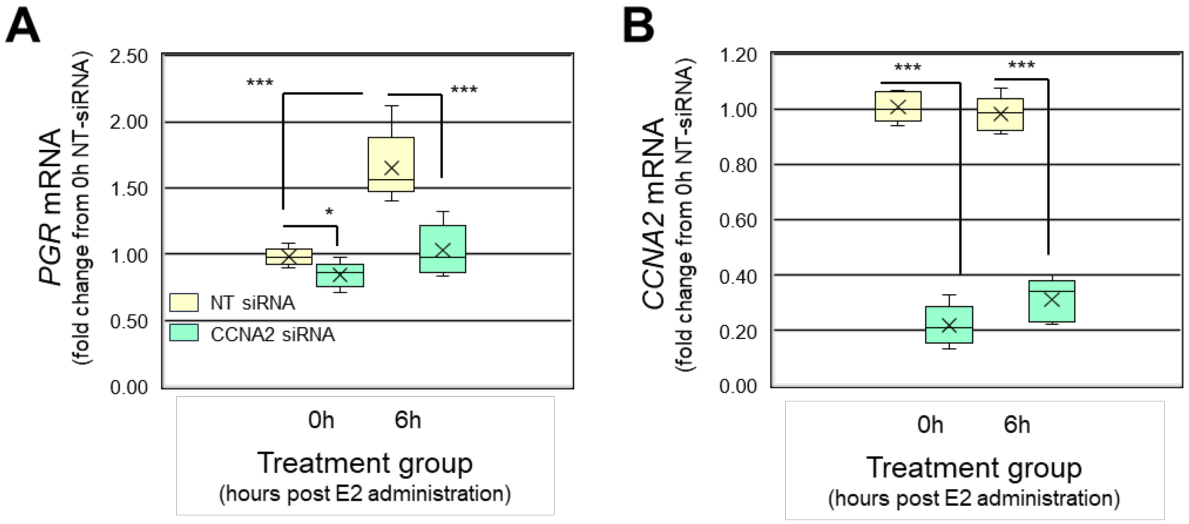

Supplemental Figure 5. Estradiol-17β in vitro induction of PGR expression in the human endometrial stromal cell line, t-HESC is compromised by CCNA2 knockdown. (**A**) qRT-PCR was performed using RNA extracted from t-HESC cells transfected with non-targeting (NT)-siRNA or CCNA2 siRNA then treated with either vehicle or 10 nM estradiol-17β for 6h and then assessed for *PGR* expression and (**B**) *CCNA2* expression. Data were analyzed by planned comparisons using two-tailed, unpaired t-tests. * = $P < 0.05$, *** = $P < 0.001$. Data are representative of five separate replicates using different cell passage numbers (n =5).

Supplemental Table 1. Top up-regulated genes in *Ccna2*$^{d/d}$ dpc 0.5 uteri.

Gene symbol	Fold change	P-value	FDR
Hoxa13	228.5633459	2.57309E-05	0.013369031
Igkv4-61	20.96814303	1.2426E-10	1.46107E-06
Krt5	14.42153088	0.000385858	0.069394932
Ano3	11.48091432	1.76881E-09	6.43317E-06
Pnoc	9.791859272	6.25768E-06	0.005007038
Sostdc1	5.666075779	2.3562E-05	0.0126022
Gm10036	5.022161102	0.000459189	0.077318106
Cav3	4.406008497	3.70156E-05	0.016026869
Tnc	4.306017077	9.70753E-07	0.00160483
Col8a1	3.926861507	0.000133506	0.034682945
Mmp13	3.853629785	3.17922E-05	0.015439928
Il12b	3.494705046	0.000445769	0.076474557
Bmp5	3.342240368	2.84865E-06	0.003047221
Serpinb11	3.206759974	0.000412249	0.071397676
Serpine1	3.129903275	0.000141503	0.035739441
Plcd4	3.060516354	6.37682E-07	0.001248624
Nipal4	2.970819396	4.76679E-06	0.004334207
Ccr7	2.965836793	9.11194E-05	0.026725921
Fgf10	2.913032032	1.56607E-06	0.002034213
Serpine2	2.795197501	1.60689E-10	1.46107E-06

Stra6	2.734479304	6.41284E-06	0.005007038
Bmp6	2.671411271	1.45543E-07	0.000343712
Crym	2.64135702	8.08156E-05	0.025338462
Ccn2	2.630355166	0.000353642	0.066603936
Cxcl13	2.517914339	3.60889E-05	0.016026869
Gm30794	2.350035022	3.62022E-05	0.016026869
Tfpi2	2.342172702	6.86623E-07	0.001248624
Timp1	2.299688709	1.98903E-06	0.002411372
Ccdc68	2.280388068	8.77251E-05	0.026588001
Cd209f	2.266262569	0.000215882	0.048283682
Ildr2	2.249918076	0.00022428	0.048553854
Nupr1	2.240499146	0.000453271	0.077034919
Fhl1	2.207145056	5.21357E-05	0.019348725
Lrrn4	2.204656149	6.74173E-05	0.022290611
Psrc1	2.17348315	4.16584E-05	0.01646864
Fosb	2.131349668	3.70294E-06	0.003741002
Synm	2.083399726	1.19073E-06	0.001676382
Inhba	2.007502149	3.85419E-05	0.016160719
Snora21	1.885685678	0.000270075	0.055810378
P2rx5	1.883845131	0.000193688	0.045371529
Nfasc	1.881103909	0.000300848	0.059466511
Bmp4	1.870909001	9.05445E-05	0.026725921

Aspn	1.867253812	0.000598633	0.097197704
Amotl2	1.845018065	6.58195E-05	0.022290611
Star	1.838892017	6.60813E-06	0.005007038
Pirb	1.836620987	0.00021686	0.048283682
Upk1b	1.826749475	0.000304865	0.059612672
G930009F23Rik	1.813583495	0.000233838	0.049445793
Phlda3	1.806564163	4.02802E-06	0.003855242
Podn	1.803764872	3.22638E-05	0.015439928
Egr2	1.801669003	8.26596E-05	0.025477378
Acat2	1.735068393	1.4563E-05	0.009458123
Myrf	1.725182627	0.00050323	0.083093074
Itga1	1.718777707	1.90481E-05	0.011177727
Apold1	1.718601956	0.000486392	0.081147134
Enc1	1.715203062	3.91021E-05	0.016160719
AB124611	1.660836018	0.000407135	0.071189861
Adam33	1.626770721	1.1984E-06	0.001676382
Cd109	1.583383472	1.90547E-05	0.011177727
Srgn	1.537145735	0.000342804	0.065619985
Dbn1	1.528443891	0.00035527	0.066603936
Plpp1	1.52047841	0.000389237	0.069394932
Cdc42ep3	1.504191194	0.000283037	0.057189098

Supplemental Table 2. Top down-regulated genes in *Ccna2*$^{d/d}$ dpc 0.5 uteri.

Gene symbol	Fold change	P-value	FDR
Dcc	-6.92122	2.09E-05	0.011542
Ovgp1	-4.55667	0.000209	0.048117
C4bp	-3.95598	0.000265	0.055363
Gm19181	-3.58923	2.53E-06	0.00288
Klf15	-2.89858	2.08E-05	0.011542
Nlrp6	-2.84518	6.92E-05	0.02248
Arl4d	-2.81501	0.000115	0.031115
Lrrc75b	-2.64622	3.68E-05	0.016027
Kcnc1	-2.47186	4.91E-05	0.018597
Cacna1e	-2.34272	0.000117	0.031305
Col6a6	-2.22427	1.51E-07	0.000344
Zfp599	-2.09059	1.33E-05	0.008953
Agmo	-2.05983	0.000141	0.035739
Sned1	-2.05738	0.000618	0.09864
Pthlh	-2.01312	0.00022	0.048284
Gria4	-1.97096	0.000299	0.059467
Gm48898	-1.96663	6.36E-06	0.005007
Homer2	-1.84314	7.54E-06	0.005484
Trpv4	-1.7217	4.59E-05	0.017769

Supplemental Table 3. Antibodies used for immunohistochemical localization.

**Antibody**	**Catalog number**	**Dilution**	**Vendor**
Cyclin A2 (CCNA2)	Ab181591	1:500	Abcam (Cambridge, MA)
Antigen Kiel 67 (Ki67)	Ab16667	1:100	Abcam
Estrogen receptor-alpha (ERα)	Ab32063	1:2500	Abcam
Phospho-ERα (ser104)	PA5-104241	1:100	Invitrogen/ThermoFisher (Waltham, MA)
Phospho-ERα (ser106)	PA5-99346	1:100	Invitrogen/ThermoFisher
Progesterone receptor A/B (PGRA/B)	8757	1:1000	Cell Signaling Technology (Danvers, MA)
Cytokeratin 8 (KRT8)	TROMA-1	5 µg/mL	Developmental Studies Hybridoma Bank (Iowa City, Iowa)
Cytokeratin 19 (KRT19)	Ab52625	1:300	Abcam
Cyclooxygenase 2 (COX2)	12375-1-AP	1:200	Proteintech (Rosemont, IL)

Supplemental Table 4. Human (h) and mouse (m) primers used for qRT-PCR.

Gene	Primer sequence (5'-3')	Accession Number
hBMP2 F	GACGCTCTTTCAATGGACGTG	NM_001200
hBMP2 R	CACCATGGTCGACCTTTAGGA	
hCCNA2 F	CGGTACTGAAGTCCGGGAAC	NM_001237
hCCNA2 R	GGTGCAACCCGTCTCGTCTT	
hIGFBP1 F	TTTACCTGCCAAACTGCAACAA	NM_000596
hIGFBP1 R	TCTTCCCATTCCAAGGGTAGAC	
hKRT18 V1 F	GAGGGCTCAGATCTTCGCAA	NM_000224
hKRT18 V1 R	AGCCCATGGATGTCGTTCTC	
hPGR F	CCTTTGGAAGGGCTACGAAGT	NM_000926
hPGR R	GAGCTCGACACAACTCCTTTTTG	
hPRL F	GAGCAAACCAAACGGCTTCT	NM_000948
hPRL R	TCAGGATGAACCTGGCTGACT	
mAqp5 F	ACAGGGCCTCTTTGGAATTAGG	NM_009701
mAqp5 R	TGAGCTTGCACTGCCTTCAC	
mBmp2 F	GCTAGATCTGTACCGCAGGC	NM_007553
mBmp2 R	CTCCACGGCTTCTTCGTGAT	
mCcna2 F	CATCTCACTACATAGCTGACTTGGA	NM_009828
mCcna2 R	GTGGCGCCTTTAATCCCAGA	
mCstb F	CGACTACTGCTGCCAAGATGA	NM_007793
mCstb R	GCTGGGACTTCACCTGGTCG	
mCtgf F	AGAACTGTGTACGGAGCGTG	NM_010217
mCtgf R	GTGCACCATCTTTGGCAGTG	
mFgf10 F	TGCGGAGCTACAATCACCTC	NM_008002
mFgf10 R	GTTATCTCCAGGACACTGTACG	
mKlf15 F	ACACCAAGAGCAGCCACCTCAA	NM_023184
mKlf15 R	GCCTTGACAACTCATCTGAGCG	
mOvgp1 F	TGCCTCAATGGGACCAGCATCT	NM_007696
mOvgp1 R	TCATAGCCAAGCCACTCCTTCC	
mPgr F	CTACTCGCTGTGCCTTACCATG	NM_008829
mPgr R	CTGGCTTTGACTCCTCAGTCCT	
mPrl3c1 F	GCCACACGATATGACCGGAA	NM_013766
mPrl3c1 R	GGTTTGGCACATCTTGGTGTT	
mPrl8a2 F	AGCCAGAAATCACTGCCACT	NM_010088
mPrl8a2 R	TGATCCATGCACCCATAAAA	

mRpl13 F	TACCAGAAAGTTTGCTTACCTGGG	NM_016738
mRpl13 R	TGCCTGTTTCCGTAACCTCAAG	
mSerpine1 F	GCACAACCCGACAGAGACAA	NM_008871
mSerpine1 R	ATGAAGGCGTCTCTTCCCAC	
mTimp1 F	GCAACTCGGACCTGGTCATAA	NM_011593.2
mTimp1 R	CGCTGGTATAAGGTGGTCTCG	
mTimp3 F	GGCTTCAGTAAGATGCCCCA	NM_011595
mTimp3 R	CTTCATACACGCGCCCTGTC	
mTnc F	CACGGCTACCACAGAGGC	NM_001369211
mTnc R	GTCCAGCAGCTTCCCAGAAT	
